# In vivo photocontrol of orexin receptors with a nanomolar light-regulated analogue of orexin-B

**DOI:** 10.1007/s00018-024-05308-x

**Published:** 2024-07-06

**Authors:** Davia Prischich, Rosalba Sortino, Alexandre Gomila-Juaneda, Carlo Matera, Salvador Guardiola, Diane Nepomuceno, Monica Varese, Pascal Bonaventure, Luis de Lecea, Ernest Giralt, Pau Gorostiza

**Affiliations:** 1 Institute for Bioengineering of Catalonia (IBEC), Barcelona Institute of Science and Technology, Barcelona, Spain; 2 Centro de Investigación Biomédica en Red – Bioingeniería, Biomateriales y Nanomedicina (CIBER-BBN), Barcelona, Spain; 3 Institute for Research in Biomedicine (IRB Barcelona), Barcelona Institute of Science and Technology, Barcelona, Spain; 4 Janssen Research & Development, LLC, San Diego, CA USA; 5 Department of Psychiatry and Behavioral Sciences, School of Medicine, Stanford University, Stanford, CA USA; 6 Wu Tsai Neurosciences Institute, Stanford University, Stanford, CA USA; 7 Department of Inorganic and Organic Chemistry, University of Barcelona (UB), Barcelona, Spain; 8 Catalan Institution for Research and Advanced Studies (ICREA), Barcelona, Spain; 9 Present Address: Department of Chemistry, Molecular Sciences Research Hub, Imperial College London, London, UK; 10 Present Address: Forschungszentrum Jülich GmbH, Jülich, Germany; 11 Present Address: Department of Pharmaceutical Sciences, University of Milan, Milan, Italy; 12 Present Address: ONA Therapeutics, Barcelona, Spain; 13 Present Address: OMAKASE Consulting, Barcelona, Spain

**Keywords:** Neuropeptide, Neuromodulation, Hypocretin, Azobenzene, Photochromism, Photoisomerization, Photopharmacology, Optopharmacology, Photoresponsive, Optical tool

## Abstract

Orexinergic neurons are critically involved in regulating arousal, wakefulness, and appetite. Their dysfunction has been associated with sleeping disorders, and non-peptide drugs are currently being developed to treat insomnia and narcolepsy. Yet, no light-regulated agents are available to reversibly control their activity. To meet this need, a photoswitchable peptide analogue of the endogenous neuroexcitatory peptide orexin-B was designed, synthesized, and tested in vitro and in vivo. This compound – photorexin – is the first photo-reversible ligand reported for orexin receptors. It allows dynamic control of activity in vitro (including almost the same efficacy as orexin-B, high nanomolar potency, and subtype selectivity to human OX_2_ receptors) and in vivo in zebrafish larvae by direct application in water. Photorexin induces dose- and light-dependent changes in locomotion and a reduction in the successive induction reflex that is associated with sleep behavior. Molecular dynamics calculations indicate that *trans* and *cis* photorexin adopt similar bent conformations and that the only discriminant between their structures and activities is the positioning of the N-terminus. This, in the case of the more active *trans* isomer, points towards the OX_2_ N-terminus and extra-cellular loop 2, a region of the receptor known to be involved in ligand binding and recognition consistent with a “message-address” system. Thus, our approach could be extended to several important families of endogenous peptides, such as endothelins, nociceptin, and dynorphins among others, that bind to their cognate receptors through a similar mechanism: a “message” domain involved in receptor activation and signal transduction, and an “address” sequence for receptor occupation and improved binding affinity.

**Supplementary Information:**

The online version contains supplementary material available at 10.1007/s00018-024-05308-x.

## Introduction

Optical methods, and optogenetics in particular, have revolutionized neuroscience by aiding our understanding of brain circuits through light-mediated monitoring and/or manipulation of neuronal activity [1–4]. Despite broad applications in terms of targeted pathways, optogenetics has several limitations [5]. While photocontrol over the exogenous light-sensitive opsins is robust, viral expression in tissues can display poor homogeneity and specificity. Moreover, the kinetics of light-gated channels differ from those of endogenous receptors and their overexpression might lead to unintended neuroplastic phenomena. Finally, irreversible optogenetic manipulations are subject to safety and regulatory requirements that hamper or slow down their therapeutic potential [6]. In recent years, photopharmacology has emerged as a complementary approach to control with light the function of endogenous proteins as well as lipids and ribonucleic acids [7, 8]. The method relies on incorporating a photoswitchable moiety into the structure of a bioactive compound and regulating the local concentration of (in)active drug through reversible illumination [9, 10]. In principle, the metabolism, pharmacokinetics, and safety of the compounds can be characterized as for conventional drugs and biologicals, in contrast to genetic manipulation methods [10]. Despite the versatility and attractiveness of the approach, many endogenous targets are yet to be addressed with synthetic photoswitches [11]. In particular, neuropeptides have received limited attention compared to small molecule synthetic ligands.

Among these, a family of neuroexcitatory peptides known as orexins (or hypocretins) [12, 13] is critically involved in regulating sleep and wakefulness [14], energy homeostasis, and reward [12]. Orexin signaling is mediated by two closely related G protein-coupled receptors (GPCRs) – OX_1_ and OX_2_ (orexin) receptors – which are strongly conserved among mammals [12, 15]. While orexin cell bodies are concentrated in the hypothalamic area [12, 13], orexin receptors are widely expressed in the central nervous system (CNS) [16, 17]. Their endogenous ligands are two peptides, named orexin-A (OX-A) and -B (OX-B), which bind to the cognate receptors with different selectivity profiles. OX-A displays high affinity towards both receptors, whereas OX-B is selective for OX_2_ [12]. In both cases, agonist activity transduces into a robust rise in intracellular calcium concentration when tested in recombinant cells expressing OX_1_ or OX_2_ [18, 19]. Small molecule antagonists are currently being developed as safe insomnia treatments [20], whereas dysfunctions of the orexinergic system have been associated with narcolepsy [21–23]. However, the discovery of non-peptide agonists to treat this and other forms of hypersomnia disorders has been challenging [24–26]. Besides, these compounds might find limited applications as orexin receptors are widely expressed in the CNS. Consequently, their activity would suffer from poor spatiotemporal selectivity and might cause adverse effects. A photocaged analogue of OX-B was recently developed to address these constraints [27], however, local diffusion limits spatial control over the release of such irreversibly light-activated ligands. In addition, byproducts released upon photouncaging might be toxic or interfere with biological processes, complicating result interpretation and eventually the use of these ligands. Lastly, photocaged compounds are difficult to store and handle, as accidental exposure to light can lead to the irreversible release of the caged molecule. Here, we present a light-regulated analogue of the orexin-B peptide, which displays similar agonist activity and can be reversibly toggled between two isomeric states characterized by different potencies at the orexin receptors in vitro. We further present preliminary results showing that photorexin allows dynamic control over endogenous OX_2_ activity in zebrafish larvae, making it one of the few photoswitchable peptides displaying activity in vivo and the first one for a CNS target. Thus, photorexin is a versatile and promising tool to modulate orexinergic transmission in research and in therapeutic contexts.

## Materials and methods

### Photorexin synthesis

Photorexin was synthesized by standard 9-fluorenylmethyloxycarbonyl (Fmoc) solid-phase peptide synthesis (SPPS) on a 0.1 mmol scale. Peptide elongation was performed using an automated microwave-assisted peptide synthesizer combined with subsequent manual elongation of the chain. The Fmoc-protected photoswitchable amino acid [3-(3-aminomethyl) phenylazo] phenylacetic acid (AMPP) was synthesized adapting a protocol published by Podewin et al. [28]. Synthesis, purification, and analytical characterization of all compounds are detailed in the Supplementary Information (SI).

### Photochromic characterization

#### Peptide quantification

After purification, lyophilized samples were reconstituted in phosphate-buffered saline (PBS, pH 7.4) and quantified by measuring the absorption of the azobenzene moiety at 323 nm with a micro-volume spectrophotometer. The peptide concentration was then determined using the Lambert-Beer law:

$$c = \frac{A}{{\epsilon \cdot l}}$$

where *c* is the calculated concentration (M), *A* is the absorbance (AU) measured at a given wavelength, *ε* is the corresponding molar extinction coefficient (25 000 M^− 1^cm^− 1^ at *λ*_323_) [29], and *l* is the optical path length (cm).

#### UV-Vis absorption spectra

Ultraviolet-Visible (UV-Vis) absorption spectra were recorded with a Shimadzu UV-1800 spectrophotometer using a far-UV quartz cuvette (Hellma, Jena, DE) with an ultra-micro cell (10 mm optical path). Samples were prepared in milliQ H_2_O at 20 µM. UV-Vis absorbance scans were performed at 25 °C in the dark-adapted state (0 min, dashed curve in Fig. S2.1) and at different irradiation times. An Ultraviolet germicidal irradiation (ARG-235-UVGI) torch, which has multiple emission lines in addition to the nominal 311 nm, was used as light source. The power density (20.8 mW∙cm^− 2^) was measured with a PM101 Power Meter Interface with USB, RS232, UART, and Analog Operation (Thorlabs GmbH), coupled to a sensor (S470C - Thermal Power Sensor Head, Volume Absorber, 0.25–10.6 μm, 100 µW − 5 W, Ø15 mm, Thorlabs GmbH) placed at 2.5 cm from the lamp and processed using Optical Power Monitor Version 4.1.4204.742 (Thorlabs GmbH).

#### Photostationary states

Identical phOX samples were obtained from a 50 µM dilution in PBS (pH 7.4, 25 °C) and were exposed to light of different wavelengths in order to study their photochromic behavior. The samples were injected for chromatographic analysis immediately after being illuminated. *Trans*→*cis* photoisomerization was studied after illumination with an ARG-235-UVGI torch light, or at 365 and 380 nm, whereas *cis*→*trans* back-isomerization was induced at 455 and 500 nm. Custom-made multi-LED dual-wavelength lamps (FC Tècnics, Barcelona, ES) were used as light sources. Light power densities were measured as previously described and ranged from 16.9 mW∙cm^− 2^ (455 nm), 2.2 mW∙cm^− 2^ (365 nm), 0.83 mW∙cm^− 2^ (500 nm) to 0.51 mW∙cm^− 2^ (380 nm).

The photostationary states were quantified integrating the area of the peaks corresponding to the *cis* and *trans* isomers in the chromatograms visualized at 220 nm. Photoisomerization kinetics were obtained by plotting the fractions of the *cis* and *trans* isomers over cumulative irradiation times. Chromatographic gradients are described in the analytical section of the SI.

#### Resistance to photofatigue

Stability to photodegradation was evaluated by exposing a phOX sample (25 µM in PBS at pH 7.4 and 25 °C) to alternating intervals (3 min each) of UV and visible light illumination (365 and 455 nm, respectively). The *trans-cis* isomeric distributions under different illumination conditions were measured by integrating the area of the corresponding peaks after UPLC analysis. These values were then converted to isomer percentages and plotted. After several cycles, no reduction could be observed in the isomers’ peak area, thus confirming photorexin stability to photodegradation.

#### Thermal relaxation

The thermal relaxation rate at 37 °C was investigated using an Infinite M200 PRO microplate reader (Tecan, Männedorf, CH). A phOX sample (50 µM in milliQ water) was placed in a 96-multiwell plate and pre-irradiated for 10 min at 365 nm. Subsequently, the plate was moved into the microplate reader and kept in the dark at 37 °C within the instrument. Data were collected every 30 min for 24.5 h and analyzed by nonlinear regression with GraphPad Prism 9. The half-life was calculated using the monoexponential decay model provided by the software.

### Photopharmacology

#### Photorexin sample preparation

Samples of *trans* phOX were kept in the dark both prior to and during the whole duration of the experiment to avoid undesired photoconversion caused by environmental light. Unless differently stated, data for *cis* phOX were obtained by pre-illuminating the samples for 10 min with 365 nm UV light.

#### PFSK-1 and CHO-K1 cell culture

PFSK-1 cells were cultured in RPMI 1640 supplemented with 10% fetal bovine serum (FBS), 100 units/ml penicillin, and 100 µg/ml streptomycin. Chinese hamster ovary-K1 (CHO-K1) cells stably transfected to express human OX_1_ were cultured in Dulbecco’s Modified Eagle’s Medium/Nutrient Mixture F-12 Ham (DMEM/F12 1:1) medium supplemented with 10% FBS and 600 µg/ml G418. All cell lines were grown at 37 °C in 5% CO_2_.

#### FLIPR Tetra calcium mobilization assay

Cells were plated at 50,000 cells/well in 96-well black-wall plates with poly-D-lysine-coated clear bottoms and grown overnight at 37 °C in 5% CO_2_. On the day of the assay, an equal volume of loading solution (2X) containing a calcium-sensitive fluorescent dye (BD Calcium Assay Kit, BD Biosciences, USA) was added to the cells and incubated for 60 min at 37 °C in 5% CO_2_. During the incubation period, 8-points half-log serial dilutions of the peptides were prepared in Hanks Balanced Salt Solution (HBSS) supplemented with 0.1% BSA starting from 345 µM stocks in milliQ water. In the assay, final concentrations of the peptides ranged from 1 µM to 0.3 nM. Calcium-mediated changes in fluorescence were monitored in a Fluorometric Imaging Plate Reader (FLIPR) Tetra instrument (Molecular Devices, USA). Fluorescence intensity was measured for 12 s to establish baseline fluorescence before addition of the test compound (20 µL/well) and further readings for a total of 155 s (excitation at 470–495 nm, detection at 515–575 nm). FLIPR parameters were set as follows: light intensity, 20%; exposure time, 0.4 s; gain, 120.

Raw data from the FLIPR Tetra was exported as the difference between maximum and minimum fluorescence observed for each well. Relative fluorescent units (RFU) were plotted against compound concentration and the curves fitted by non-linear regression using GraphPad Prism. Results are from two independent experiments each run in triplicates. Agonist potencies (EC_50_) are expressed as means + SD.

#### TsA201 cell culture

Transformed human kidney tsA201 cells were purchased from the European Collection of Authenticated Cell Culture. Cells were maintained at 37 °C in a humidified atmosphere with 5% CO_2_ and grown in Dulbecco’s Modified Eagle’s Medium/Nutrient Mixture F-12 Ham (DMEM/F12 1:1) medium (Life Technologies), supplemented with 10% FBS (Life Technologies) and 1% penicillin/streptomycin (Sigma-Aldrich).

#### Cell transfection

Clones of GFP-labelled orexin receptors were kindly provided by Prof. Jyrki Kukkonen and the exact protein sequences have been reported by Putula et al. [30]. The plasmid for the genetically encoded calcium indicator R-GECO1 was acquired from Addgene. Cells were transiently transfected with hOX_1_ or hOX_2_ receptors and R-GECO1 with X-tremeGENE 9 DNA Transfection Reagent (Roche Applied Science) following the manufacturer’s instructions. After 24 h, cells were harvested with accutase (Sigma-Aldrich) and plated onto 16-mm glass coverslips (Fisher Scientific) pre-treated with poly-L-Lysine (Sigma-Aldrich) to allow cell adhesion. Experiments were performed using pre-confluent cultures between 48 and 72 h after transfection.

#### In vitro single-cell calcium imaging

The buffer used for single-cell intracellular calcium recordings contained: 140 mM NaCl, 5.4 mM KCl, 1 mM MgCl_2_, 10 mM HEPES, 10 mM glucose and 2 mM CaCl_2_ (pH 7.4). Before each experiment, cells were mounted on the recording chamber (Open Diamond Bath Imaging Chamber for Round Coverslips from Warner Instruments, Holliston, USA) and rinsed with fresh solution. The recording chamber was filled with 1 ml buffer and placed on an IX71 inverted microscope (Olympus, Tokyo, JP) with a XLUMPLFLN 20XW x20/1 water immersion objective (Olympus). R-GECO1 was excited during 50 ms at 562 nm by using a Polychrome V light source (Till Photonics, Kaufbeuren, DE) equipped with a Xenon Short Arc lamp (Ushio, Oude Meer, NL) and a 585-nm dichroic beam splitter (Chroma Technology, USA). Emission at 600 nm was filtered by an ET 630/75 nm emission filter (Chroma Technology) and finally collected by a C9100-13 EM-CCD camera (Hamamatsu). The compound was applied during imaging acquisition by pipetting a small volume into the accessory pool of the recording chamber. The final dilution was approximately 1:1000.

Photostimulation during recordings was performed illuminating the entire focused field using the Polychrome V connected to a personal computer. Shutter and wavelength were controlled using the Patchmaster software (HEKA, DE). Light intervals lasted a total of 5 min for all the experiments with flashes of blue (455 nm, 3.5-seconds duration) and UV (365 nm, 3.5-seconds duration) light. Images were acquired at room temperature at a 4-s interval using the SmartLux software (HEKA).

Images were analyzed using FIJI (ImageJ). Data were analyzed and processed using GraphPad Prism v8.3.1. Statistical differences were determined by one-way ANOVA followed by Tukey’s multiple comparison *post-hoc* test. A *p*-value ≤ 0.05 was considered statistically significant.

#### Animal housing

Wild-type zebrafish embryos (Tüpfel long-fin strain) were purchased from the animal facility of the Barcelona Biomedical Research Park (PRBB) and raised in darkness for 7 days at 28.5 °C in UV-sterilized tap water. Petri dishes housing the animals were cleaned and replenished with fresh water on a daily basis. Animal development was checked every 24 h. Unhealthy or abnormal embryos and larvae were removed and euthanatized in tricaine methanosulfonate 0.02%. All experiments and procedures were conducted according to the European Directive 2010/63/EU.

#### General methods for locomotor assays

All photorexin solutions were freshly prepared prior to any experiment from 5 mM stock solutions and stored in the dark at -20 °C. The stock solutions were diluted with UV-sterilized water to reach the desired final concentrations. Larvae were recorded and video analyzed using the Zebrabox and Zebralab software (ViewPoint Life Sciences, Lyon, FR). Briefly, larvae at 7 days postfertilization (dpf) were randomly distributed over the different treatment groups. Each individual was placed in a well of a 96-well plate containing 200 µL of fresh UV-sterilized water per well. Each treatment included 12 specimens. The larvae were left undisturbed for 40 min in the dark. Continuously, 100 µL of water was removed from each well and replaced with a double-concentrated treatment solution before video recording began. Photorexin solutions were added with a multichannel pipette to exclude differences due to delays in the application of each dose. For the first 20 min, larvae were kept in darkness to measure their basal activity, named resting period (RP). Subsequently, 365 nm and 455 nm light cycles were applied to photoisomerize the peptide between its respective *cis* and *trans* photostationary states. Illumination periods at the beforementioned wavelengths lasted for 1, 2, and 5 min (plotted with purple and blue background areas to indicate 365 nm and 455 nm light, respectively).

Illumination was performed at each wavelength with built-in arrays of 12 light-emitting diodes (LEDs) placed 12 cm away from the 96-well plate. Light intensities, measured with an optical power meter (model Newport 1916-C from Newport Corporation, Irvine, USA), were 0.59 mW·cm^− 2^ for 365 nm (UV light) and 0.24 mW·cm^− 2^ for 455 nm (visible blue light). All experimental procedures were performed at 12:00 pm (CET).

#### Tracking and analysis of swimming activity

Tracking of each individual was performed in real time and the acquired data was integrated using 1-, 2- or 5-minute intervals with the Zebralab software (ViewPoint Life Science). Locomotor activity was measured as the sum of all the swimming distances during burst activities (movements faster than 6 mm·s^− 1^) and reported as millimeters swum during 1-minute intervals. Statistical analysis was performed using GraphPad Prism 9. Results are represented as means ± SEM. Statistical differences were determined by two-way ANOVA with Tukey’s or Dunnett’s multiple comparison test. *P*-values less than 0.05 were considered to indicate statistically significant differences. Further details can be found in the figure captions.

### Structural characterization

#### Circular dichroism

The solution structure of photorexin was investigated by circular dichroism (CD) spectroscopy to assess the impact of the photoswitchable amino acid on the secondary structure of the peptide. PhOX was dissolved in PBS (pH 7.4) – with or without 30% 2,2,2-trifluoroethanol (TFE) – at a final concentration of 25 µM. Spectra were recorded on a J-815 spectrophotometer (Jasco, Easton, USA) in the dark-adapted state or after exposure of the samples to UV light (10 min illumination at 365 nm). Absorbance was measured in a quartz cuvette (0.1 cm optical path) at 28 °C. Scan speed was set at 100 nm/min, with a 1.0-nm bandwidth and a 1-s response time. For baseline correction, spectra of the pure buffers were also recorded. All high-tension voltage traces were below 900 V between 190 and 300 nm. Molar ellipticity (deg·cm^2^·dmol^−1^) values were calculated from experimental ellipticity using the following equation:

$$\left[ \theta \right] = \frac{{\left[ {\theta {\rm{exp}}} \right] \cdot {{10}^6}}}{{10 \cdot l \cdot c}}$$

where [*θ*exp] is the measured ellipticity (mdeg), *l* is the length of the optical path (cm), *c* is the concentration of the peptide (µM). Spectra were then normalized into mean residue ellipticity (MRE) dividing molar ellipticity values by the number of residues in the peptide. The percentage of α-helicity was calculated knowing that the MRE of a 100% helical peptide at 222 nm measures:

$$\left[ {{\theta _{{\rm{222}}}}} \right] = 40000 \cdot \frac{{n - 4}}{n}$$

where *n* is the number of residues [31]. The recorded spectra were processed with OriginPro 8.5 (Origin Lab, Northampton, USA) and smoothed with a 21-points Savitzky-Golay filter before being plotted using GraphPad Prism 9.

#### Replica exchange molecular dynamics (REMD)

Replica exchange with solute tempering (REST) molecular dynamics were performed to assess the structures of all the peptides while in solution. Atom coordinates for the native OX-B peptide were extracted from the Protein Data Bank (PDB ID: 1CQ0), and the structure was manually modified to yield the desired truncated orexin analogue (residues 6–28, C-terminal carboxamide). For the photoswitchable analogues, the atom coordinates for the azobenzene moiety were extracted from PDB ID: 2H4B, a solution NMR-solved structure of a *cis*-AMPP peptide. This moiety was then inserted into the appropriate position of the orexin peptide scaffold and a short minimization was performed to relieve local clashes (restraints were imposed on the *cis* isomer). To obtain the initial coordinates of the *trans* azobenzene peptide, a short unrestrained molecular dynamics simulation was performed to relax the azo bond from *cis* to *trans.* All peptides were then solvated using the SPC explicit water model and 10 REST replicas were performed with the Desmond-2018 software (Schrödinger, USA) at temperatures ranging from 300 to 522 K. The NPT ensemble was employed, and frames were collected every 20 ps for a total of 20 ns for each replica, thus accounting for a total of 200 ns simulation time for each peptide. The first replica (at 300 K) of each simulation was inspected, analyzed and representative clustering of all frames was performed to assess the most populated structures.

## Results and discussion

**Fig. 1:**
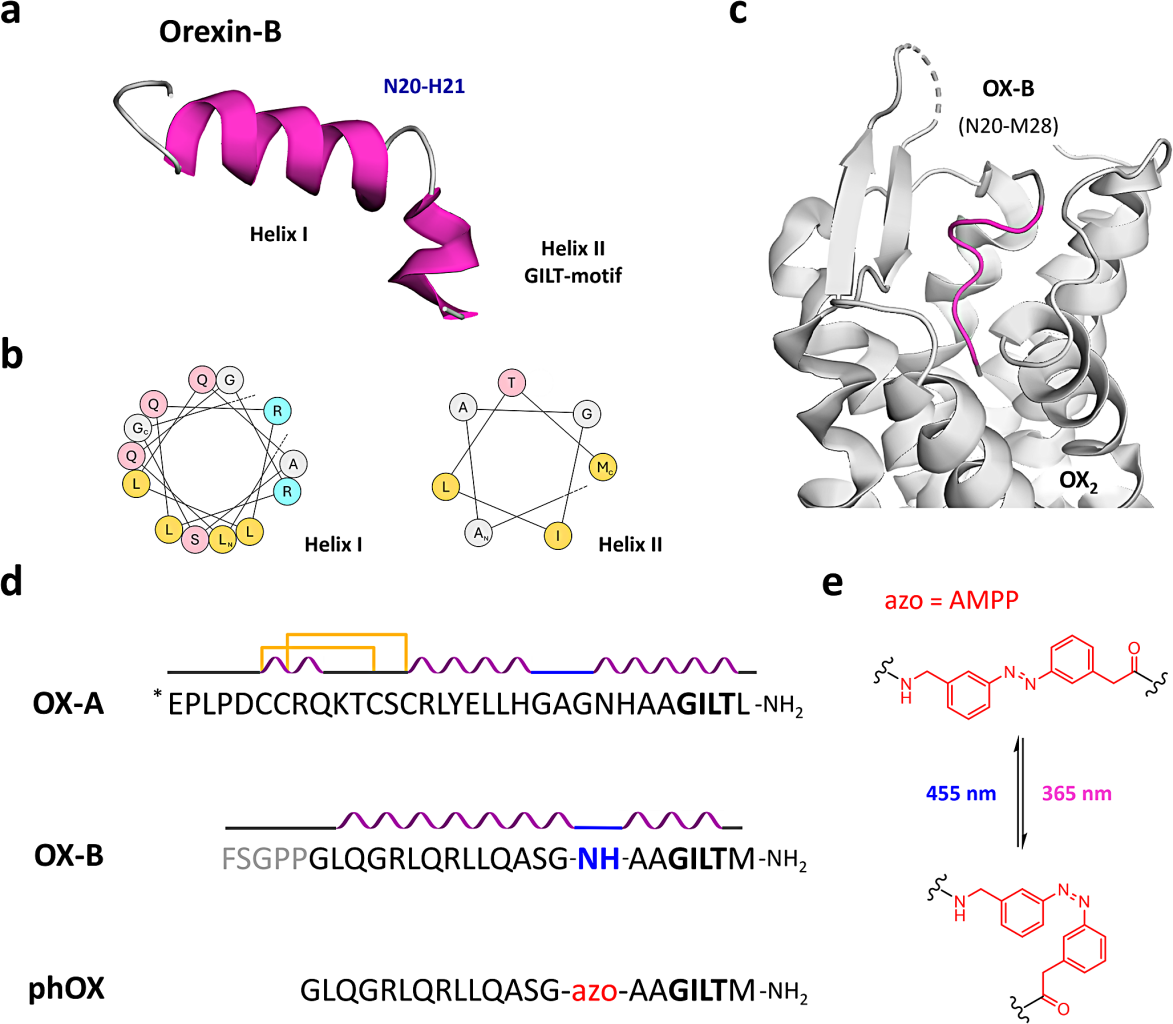
Rational design of photorexin (phOX) (**a**) NMR-derived aqueous solution structure of the orexin-B peptide (PDB: 1CQ0). A flexible linker (N20 and H21) is located between Helix I (L7-G19) and the biologically active GILT-motif in Helix II (A23-M28). (**b**) Helical wheel projections of OX-B Helix I (L7-G19, left) and Helix II (A23-M28, right). Aliphatic residues are represented in yellow, polar residues are in pink, positively charged residues are in cyan, and small residues are in grey. The N- and C-termini of each helix are indicated with N or C, respectively. (**c**) Structure of OX-B (residues N20-M28) bound to OX_2_ in an extended conformation as determined by single-particle cryo-electron microscopy (PDB: 7L1U). The peptide C-terminus is anchored deep into the receptor binding pocket with flexible residues N20-H21 exposed to the extracellular surface. OX-B GILT-motif is represented in purple, whereas other residues are in grey. (**d**) Comparison of the amino acid sequences and secondary structures of orexin-A (OX-A), orexin-B (OX-B), and photorexin (phOX). In the latter, the unstructured N-terminus (in grey in OX-B) was removed, while the flexible linker (N20 and H21, in blue in OX-B) was replaced with a photoswitchable amino acid (azo, in red in phOX). *E represents pyroglutamic acid, C-termini are amidated and indicated as -NH_2_ at the end of the sequences. In the scheme, α-helices are pictured in purple, turns are shown in blue, and intramolecular disulfide bridges as yellow lines. (**e**) *Trans* and *cis* structures of the photoswitchable ω-amino acid [3-(3-aminomethyl)phenylazo]phenylacetic acid (AMPP, in red)

As revealed by two-dimensional nuclear magnetic resonance (2D NMR) spectroscopy, both orexin isoforms present relatively high helical content in their aqueous solution state [32–34]. OX-B comprises two α-helices spanning residues Leu7-Gly19 (helix I) and Ala23-Met28 (helix II), respectively. A flexible linker (residues Asn20 and His21) connects them orienting their axis about 60–80° relative to one another (PDB ID:1CQ0) (Fig. 1a-b) [32]. OX-A shows similar structural features to OX-B, however, its N-terminus contains a third helix located between residues Cys6-Gln9 (helix III) and conformationally restrained by two disulfide bridges (PDB ID:1WSO) (Fig. 1d) [34]. 

So far, stapling with a photoswitchable crosslinker has enabled light-regulation over the secondary structure of helical peptides to control a diversity of cellular processes [35–40]. However, this methodology, which relies on the formation of a bisthioether between a photoswitch and two strategically site-mutated Cys residues, is complicated by the presence of disulfide bonds in the peptide sequence [41]. Unfortunately, previous studies show that removing OX-A disulfide bonds by reduction or by isosteric replacement of the Cys residues can lead to a nearly 10-fold loss in agonist activity towards both orexin receptors [42–44]. In addition, shortening the OX-A N-terminus also causes a reduction in peptide activity [44, 45]. Thus, based on these considerations, in our proof of concept demonstration we decided to focus on OX-B structure to minimize the structural and synthetic complexity, and the impact of chemical modifications on biological activity.

Recently, Hong et al. determined the activated-state structure of OX_2_ by cryogenic-electron microscopy (cryo-EM) and demonstrated that OX-B C-terminus (helix II in the aqueous solution structure) is anchored deep into the receptor binding pocket [46]. In contrast, residues corresponding to the flexible linker (Asn20-His21) are exposed towards the receptor extracellular surface (Fig. 1c). The cryo-EM density map also shows a less-defined density feature, whose shape and location suggest that the N-terminus of the peptide interacts via an α-helical structure with the extra-cellular loop (ECL) 2 and N-terminus of OX_2_. The formation of a similar hydrophobic interface has been described between OX-A and both orexin receptors and its involvement in receptor activation is supported by mutagenesis studies and molecular dynamics (MD) simulations [47, 48]. Surprisingly, Hong and coworkers also found that OX-B C-terminal segment binds to the receptor in an extended conformation rather than adopting a helical structure (Fig. 1c) [46]. Considering both the technical hurdles and the limited impact of photoregulating orexin helicity [49], we reasoned that replacing the flexible hinge of OX-B with a photoswitchable amino acid might allow to reversibly disrupt the overall geometry of the ligand.

Consequently, we designed a photoswitchable analogue of orexin-B as follows: (i) Asn20 and His21 were replaced by an ω-amino acid containing an azobenzene moiety; (ii) the linear sequence of the peptide was reduced to the minimum required for receptor activation (OX-B_6-28_) by removing the N-terminus; and (iii) the C-terminal amidation was maintained as beneficial for both peptide activity and stability (Fig. 1d). As molecular switch, we chose [3-(3-aminomethyl)phenylazo]phenylacetic acid (AMPP) (Fig. 1e). This choice took into account the excellent photochromic properties of azobenzene, the possibility of obtaining great alterations in geometry upon photoisomerization, and several successful reports where the switch component had been inserted into β-turns and within α-helices to photoregulate the orientation of the associated secondary structures [28, 50–53]. In addition, the two methylene bridges adjacent to the azobenzene core could guarantee enough flexibility to the system [54]. 

**Fig. 2:**
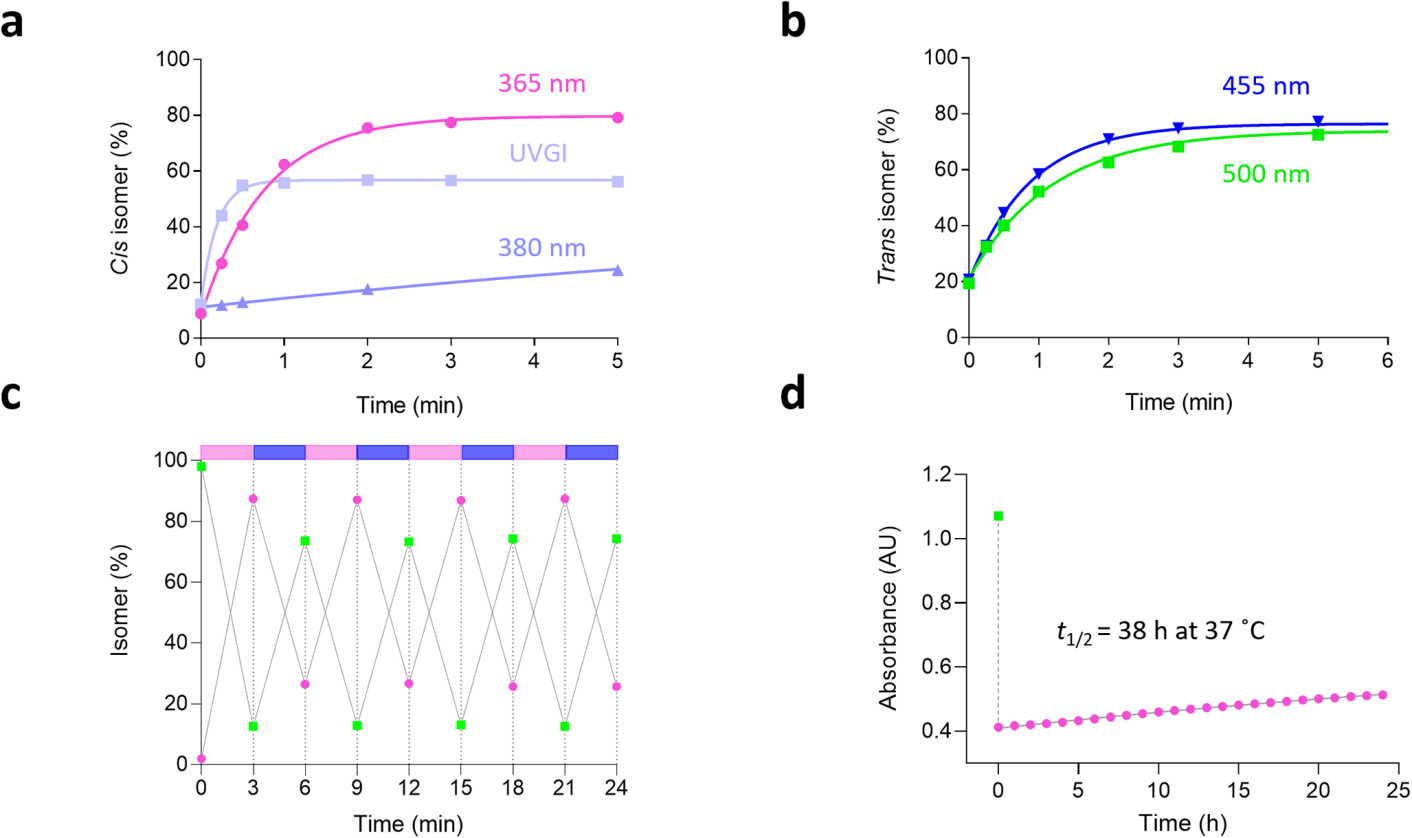
Photorexin photochromic characterization. (**a**-**b**) Isomeric distribution of a phOX sample (50 µM in PBS at pH 7.4 and 25 °C) when photoswitching from *tran*s to *cis* (**a**) or from *cis* to *trans* (**b**) after cumulative illumination at different wavelengths. Percentages of the two species were quantitatively determined by UPLC analysis upon resolving the peaks of the two isomers at given illumination times. Data were fitted to a monoexponential decay model. (**c**) Reversibility and stability of the photochromic behavior of phOX (25 µM in PBS at pH 7.4 and 25 °C) over several cycles (3 min each) of photoinduced isomerization at 365 nm (*trans→cis*) and 455 nm (*cis→trans*). Percentages of the two isomers were determined by UPLC analysis after each cycle. (**d**) Thermal relaxation of phOX at 37 °C in PBS (pH 7.4) as monitored by UV-Vis spectroscopy at 325 nm. Data were fitted to a monoexponential decay model for *cis* half-life determination (*t*_1/2_ = 38 h)

Fmoc-protected AMPP was synthesized as reported in the literature and incorporated into the backbone of photorexin combining automated and manual solid-phase peptide synthesis (SPPS) with a standard Fmoc-based strategy [28]. Insertion of the azo-amino acid was confirmed by UV-Vis spectroscopy (Fig. S2.1). Furthermore, taking advantage of the thermal bistability reported for this azobenzene [52], the photochromism of photorexin could be characterized by UPLC analysis. Thus, the percentages of the two isomers were quantified while testing a variety of illumination conditions. In particular, we studied switching kinetics and photostationary states using a UVGI torch light at our disposal as well as 365 and 380 nm UV light for the *trans*→*cis* photoisomerization (Fig. 2a); and 455 and 500 nm visible light (blue and green, respectively) for the reverse process (*cis*→*trans*) (Fig. 2b).

*Trans* photorexin could be readily converted to the *cis* isomer by illuminating between 311 and 365 nm, whereas the extent of photoisomerization was lower at longer wavelengths (e.g. 380 nm). From an initial photostationary state of 13% *cis* and 87% *trans* under benchtop conditions, an excellent 20:80 (*trans:cis*) ratio was achieved after 2 min of illumination at 365 nm (Fig. 2a). In contrast, exposure to blue light for 3 min back-isomerized the compound and reverted the ratio to 74:26 (*trans:cis*). Similar values could also be reached by illuminating with green light for a longer time (~ 5 min) (Fig. 2b).

We then tested the photochromic stability of our peptide. Photorexin could be readily toggled between 87% *cis* and 74% *trans* with alternating cycles of UV and blue light of 3 min each. The process was repeated several times without significant degradation or bleaching (Fig. 2c and Fig. S2.2). Finally, the thermal relaxation of the *cis* isomer (*t*_1/2_ ~ 38 h at 37 °C) was determined as displayed in Fig. 2d.

It must be noted that although UV wavelengths required to photoswitch our peptide do not penetrate deep into tissue, symmetric azobenzenes like AMPP can also be isomerized with mid-infrared light using three-photon excitation. Multiphoton (two- and three-photon) excitation offers deep penetration and three-dimensional focusing at the microscale [55]. 

Photorexin pharmacodynamics were then examined to understand: (i) how replacement of the flexible hinge with the photoswitchable AMPP amino acid affected both potency and maximal efficacy of the peptide; and (ii) to which extent these properties could be photoregulated. Given the convenient bistability of the two isomers, pre-illuminated samples were tested using an in vitro functional assay that monitors real-time intracellular calcium responses through a Fluorometric Imaging Plate Reader (FLIPR Tetra®).

The activity was evaluated either in CHO-K1 cells stably transfected with the human OX_1_ receptor or in PFSK-1 cells. The latter is a human neuroectodermal cell line, derived from a brain tumor, that innately expresses the hOX_2_ receptor [56]. Both orexin-B and our photoswitchable analogue increased calcium release in a concentration-dependent manner, unambiguously indicating agonist activity (Fig. S3.2). The potency of the native peptide was consistent with the reported values [12, 44]. Promisingly, photorexin displayed almost the same efficacy as OX-B and high nanomolar potency towards both receptor subtypes. The EC_50_ of dark-adapted photorexin (*trans*-enriched) at hOX_2_ was only 4-fold shifted to the right compared to the dose-response curve of the endogenous peptide. This reduction in potency was slightly higher at hOX_1_, where *trans*-enriched photorexin displayed a 9-fold decrease in activity (Table S3.1).

Despite modest light-induced changes, dark-adapted photorexin consistently exhibited statistically significant higher potency compared to the *cis*-enriched peptide (1.4-fold and 2.0-fold at OX_1_ and OX_2_, respectively). As the calcium-sensitive dye used in the assay requires 470–495 nm excitation, blue light was likely to back-isomerize pre-illuminated photorexin (*cis*-enriched) to mainly *trans*, thereby reducing any difference in activity between the two samples. To overcome this limitation, we decided to assess intracellular calcium responses in a different system.

Calcium fluorescence imaging also allows the quantification of real-time changes in cytosolic calcium concentration but with single-cell or subcellular resolution. These experiments were performed in a line of transformed human kidney cells - tsA201 - expressing either OX_1_ or OX_2_ along with R-GECO1, a genetically encoded calcium indicator sensitive to green light excitation (λ_ex_ = 562 nm). In addition to overcoming the aforementioned limitations of blue light excitation, R-GECO1 red-shifted absorption spectrum offered the possibility of photoisomerizing the peptide in situ, which proved advantageous compared to testing only pre-irradiated samples.

**Fig. 3:**
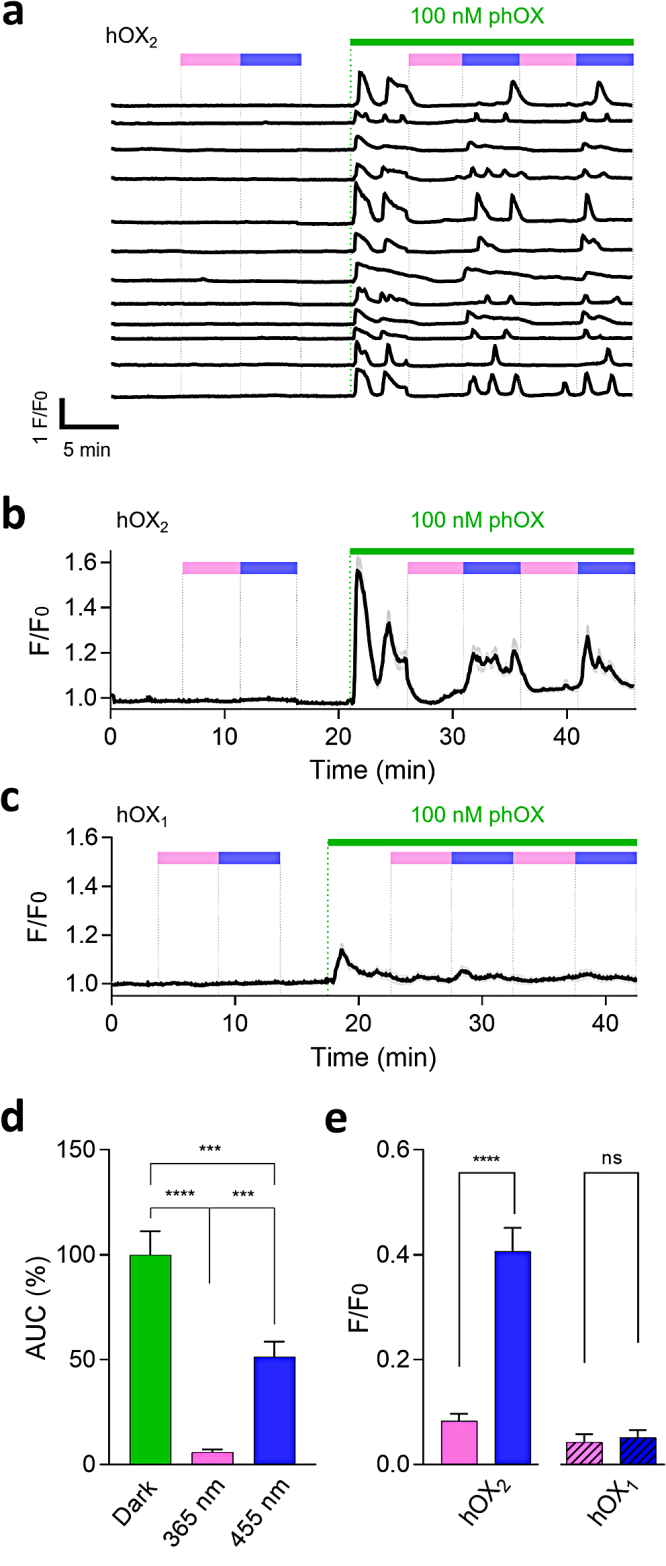
Photorexin pharmacodynamics (**a**) Representative single-cell calcium traces (*n* = 12) in tsA201 cells transiently expressing GFP-hOX_2_ and R-GECO1. Upon application of 100 nM photorexin (green bar) in its dark-adapted state (*trans*, white area) cells gave sharp responses. Calcium oscillations were then abolished upon exposure to cycles of UV light (365 nm, *cis*-enriched state in pink) and recovered with blue illumination (455 nm, *trans*-enriched state in blue). (**b**) Real-time intracellular calcium recordings (averaged traces, black line, *n* = 20) from tsA201 cells co-expressing hOX_2_ and R-GECO1 upon application of 100 nM photorexin (green bar). Traces were recorded in the dark (white area) and under cycles of illumination with UV (365 nm, pink) and blue (455 nm, blue) light. Grey area represents ± SEM. (**c**) Real-time calcium imaging responses (averaged traces, black line, *n* = 20 cells) from tsA201 cells co-expressing hOX_1_ and R-GECO1. Application of photorexin (100 nM, green bar) evokes mild responses in the dark and under blue light. Grey area represent ± SEM. (**d**) Efficacy of phOX (100 nM) on tsA201-hOX_2_ quantified in the dark (green bar) and after subsequent illumination with 365 nm UV light (pink bar, *cis* enriched) and back-isomerization to *trans* with 455 nm blue light (blue bar). Responses were quantified as Area Under the Curve (AUC) and normalized to those obtained with dark-adapted phOX. Data represent means ± SEM (*n* = 20 cells). Statistical differences were determined by one-way ANOVA with Tukey’s multiple comparison test (***, *p*-value ≤ 0.001; ****, *p*-value ≤ 0.0001). (**e**) Selectivity of phOX (100 nM) over tsA201-hOX_2_ (full-colored bars) and tsA201-hOX_1_ (dashed bars) compared after illumination with 365 nm UV light (*cis* enriched, in pink) and after back-isomerization to *trans* with 455 nm blue light (in blue). Data represent amplitude means ± SEM (*n* = 20 cells). Statistical differences were determined by one-way ANOVA with paired-sample Wilcoxon signed-rank test (ns, not significant; ****, *p*-value ≤ 0.0001)

Photorexin was first applied as a control to tsA201 cells transfected only with the calcium indicator. The absence of response to the compound and concomitant UV or blue illumination ruled out light-induced artifacts due to non-specific R-GECO1 stimulation (Fig. S3.3). Subsequently, 100 nM photorexin was applied to cells co-transfected with R-GECO1 and GFP-labelled hOX_2_. The peptide evoked a sharp transient response in the dark, displaying oscillations with a period of 1–3 min depending on each cell. Illumination with UV light terminated these responses in a few seconds, and oscillatory activity was resumed upon exposure to blue light (Fig. 3a-b). Similar patterns of calcium signals could be reproduced in at least two cycles of alternating UV and visible light as well as at lower doses (30 nM, Fig. S3.4), thus demonstrating that photorexin can robustly and reversibly regulate the activity of orexin receptors with light and that it behaves as a *trans*-active agonist with nanomolar potency. The dynamic photocontrol of orexin receptor activity enabled by photorexin is in contrast to the irreversible photorelease of caged OX-B demonstrated in vitro [27]. In hOX_1_-expressing cells, the dark-adapted peptide also produced an increase in calcium influx, although smaller in magnitude than in tsA201-hOX_2_. UV- and blue-light illumination (365 and 455 nm, respectively) elicited no effect (Fig. 3c and Fig. S3.5).

Calcium traces from different cells were then integrated to quantify the observed responses. Noticeably, UV-illuminated photorexin (*cis*-enriched) retained only 6% of the dark-adapted peptide activity towards hOX_2_. While illumination with blue light rapidly restored calcium mobilization, recovery was partial (52% of the initial responses) probably owing to receptor desensitization after prolonged agonist exposure (Fig. 3d). In the dose-response curves, dark-adapted photorexin displayed significantly lower potency towards hOX_1_. In contrast, both the UV- and blue-light illuminated forms evoked no response on this receptor, thus demonstrating selectivity towards hOX_2_ (Fig. S3.5 and Fig. 3e). In light of the promising results obtained through calcium fluorescence, we moved on to test photorexin activity in vivo.

The orexin neural network in zebrafish has been studied in detail and, as in mammals, it is involved in many physiological functions including sleep/wake cycles, homeostasis, feeding, and locomotor activity [57–62]. Between 16 and 60 neurons compose the zebrafish’s network with a similar gene to mammals encoding for two orexins neuropeptides (OX-A/-B) [61]. Only one orexin receptor has been found in zebrafish, closely related to mammalian OX_2_ (70%). Its binding pocket is highly conserved throughout species [15, 61]. The simplicity of the network in zebrafish and its close resemblance to higher mammals, along with the advantages of using an established neuropharmacological [63–66] and photopharmacological [7, 67] animal model, allowed us to investigate the effects of photorexin on locomotor activities of wildtype larvae.

**Fig. 4:**
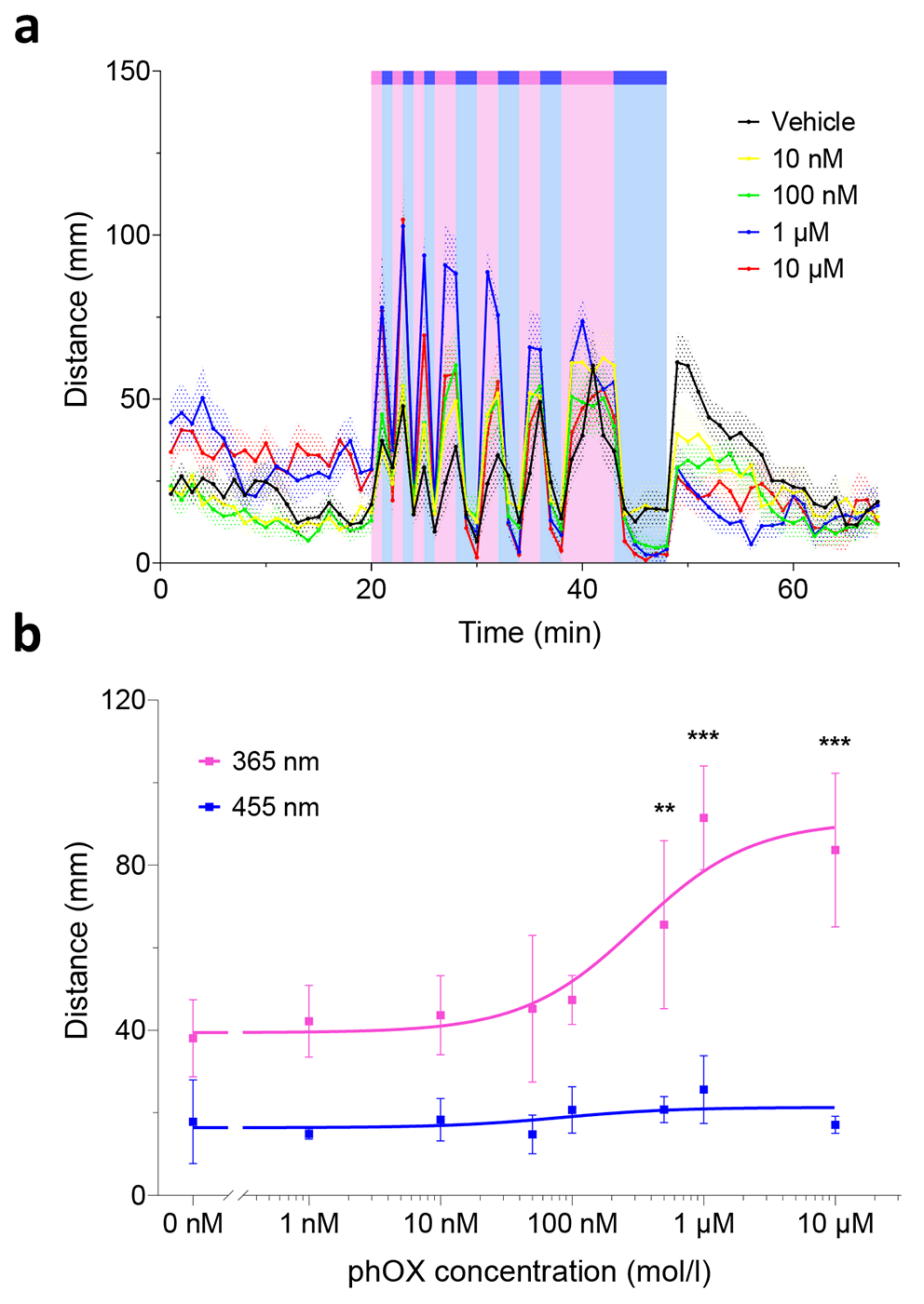
Photorexin in vivo activity in wildtype zebrafish (**a**) Fast-swimming distances (faster than 6 mm·s^− 1^) are plotted for larvae treated with different doses of phOX (vehicle – black, 10 nM – yellow, 100 nM – green, 1 µM – blue, and 10 µM – red traces). Distances were integrated over 1-min intervals. UV illumination periods increased the swimming activity of larvae exposed to high phOX doses (1 and 10 µM), while blue light reversed activity to levels of non-treated larvae. This different behavior was maintained over several light cycles. After the last illumination interval (5 min at 455 nm), all treated larvae showed a weaker induction response (i.e., the fast increase and slow decrease in locomotion observed upon switching off visible light). Patterned areas represent ± SEM (*n* = 12 larvae per condition). (**b**) Dose-response curves (distance swum in mm) in zebrafish larvae of *cis* (365 nm, in purple) and *trans* (455 nm, in blue) phOX upon integrating the 3 consecutive 1-min illumination intervals between minutes 20 and 26 for each wavelength (365 nm and 455 nm, purple and blue traces respectively). Data at 0 nM corresponds to the vehicle under both illumination conditions. Error bars represent ± SD (*n* = 12 larvae per treatment group). Solid traces are fitted to a log(agonist) vs. response model. Statistical differences were determined by two-way ANOVA with Tukey’s multiple comparison test (**, *p*-value ≤ 0.01; ***, *p*-value ≤ 0.001)

Individual larvae were placed in separate wells of a 96-well plate, each containing increasing concentrations of photorexin. Each individual was tracked and monitored over 68 min under different illumination conditions, alternating between 365 nm, 455 nm, and dark periods (respectively indicated by pink, blue, and no background in Fig. 4a). The time courses of 12 larvae per concentration group integrated every minute are shown in Fig. 4a. All applied concentrations are shown in Fig. 4b (0–10 µM).

High doses of photorexin (1 and 10 µM) significantly enhanced locomotion (distance swum over 1-min integration intervals) under 365 nm light compared to non-treated larvae, for all UV-illuminated periods (three 1-min bouts at 20, 22, 24 min; three 2-min bouts at 26, 30, 34, and one 4-min bout at 38 min). This increase in activity was sharply reverted under visible blue light and all treated larvae swam distances similar to non-treated individuals (three 1-min bouts at 21, 23, 25 min; three 2-min bouts at 28, 32, 36, and one 4-min bout at 42 min). Interestingly, the successive induction reflex (i.e., the fast increase and slow decrease in locomotion observed upon switching off visible light) [68, 69] is significantly reduced for all photorexin-treated groups in comparison to controls as observed at t = 49 min (Fig. 4a). These preliminary findings are consistent with sleep behaviors in zebrafish after exposure to orexin agonists, which elicit lower responsiveness upon light-to-dark changes [60]. Several photoswitchable small molecules with µM-mM potencies [70–73] and photoswitchable peptides with 1-100 µM potencies [74] are active in zebrafish by simple addition in water. Therefore, it is not surprising that photorexin, which displays low-nM potencies in vitro, readily enables photocontrol of behavior at sub-µM concentrations (500 nM, Fig. 4b) in vivo. Photoswitchable small molecules targeting the CNS are absorbed by the skin, gills, and pumping system, and produce behavioral effects in about 20 min [70–73]. While OX-A has been shown to penetrate the blood-brain barrier by simple diffusion after intravenous administration or blood-free perfusion in mammals [75–77], OX-B is not as lipophilic and is readily metabolized [75]. Shortening a peptide sequence and replacing polar residues with a hydrophobic moiety could generally benefit permeability, however, the chemical modifications introduced into the structure of photorexin are unlikely to alter significantly the overall physicochemical properties of the molecule compared to the parent compound. Thus, the effects observed in zebrafish might originate from intranasal delivery to the CNS [78–80] and/or result from the activation of peripheral orexin receptors expressed in the gastrointestinal tract [81–84]. 

It must be noted that rodents have been the model of choice for developing hypnotics and psychostimulants despite their different sleep patterns from humans. As an alternative, zebrafish larvae show peak daytime activity and rest at night, are cost-effective, and are amenable to high-throughput screening as shown in Fig. 4. Thus, photorexin is an in vivo neuroactive photoswitchable peptide that holds high relevance to study physiology and to develop future phototherapies.

Overall, both in vitro and in vivo results validate the rationale behind our photoswitchable peptide ligand design. While interventions at the flexible hinge are compatible with preserving the functional activity of the peptide, peptide ligands require proper orientation of the axis to ensure receptor agonism and intracellular signaling. This achievement offers the ability to control endogenous orexin receptors remotely and in the different regions of the brain that receive orexinergic projections, and allows reversible and repeatable activation of orexin receptors at high temporal resolution to investigate gating mechanisms. To examine this question, we further studied photorexin structures associated with the observed differences in receptor activity.

In order to correlate the functional potencies of the *trans* and *cis* isomers with their respective conformations, we performed circular dichroism (CD) studies and molecular dynamics calculations. Photorexin CD spectra in phosphate buffer saline displayed a strong positive band at 195 nm and a negative one at 218 nm (Fig. S4.1A). This behavior suggests the presence of a β-strand conformation. The measurements were then repeated in the presence of trifluoroethanol (TFE), a cosolvent that creates a more hydrophobic environment and promotes helicogenic intramolecular interactions. Similarly to what had been reported for the native OX-B, the addition of 30% TFE enhanced the peptide helicity (40%) as indicated by a negative band at 207 nm and a shoulder at 220 nm (Fig. S4.1B) [32, 43]. These results confirmed that the replacement of the flexible linker with a photoswitchable moiety did not prevent the peptide from adopting a helical conformation. Interestingly, while in 30% TFE photorexin helicity was not affected by light-induced photoisomerization of AMPP, the CD spectrum recorded in PBS displayed a loss in secondary structure after exposure to UV light. Despite the variation being mild, we investigated if the detected structural changes were restricted to photorexin helix I or helix II and could partially account for the loss of potency of the *cis* isomer.

We thus performed replica exchange with solute tempering (REST) molecular dynamics simulations in explicit water to analyze the secondary structures of *trans* and *cis* photorexin and to relate them to their closest non-photoswitchable analogue (truncated OX-B, residues 6–28, with no amino acid replacement at the flexible hinge). Atom coordinates were extracted from the solution structure reported by Lee et al. (PDB ID: 1CQ0) [32] and we manually modified them to truncate the N-terminus and, in the case of our analogues, insert those of NMR-solved *trans* and *cis* AMPP (PDB ID: 2H4B) [85]. As an indicator of helicity, the average number of backbone H-bonds formed during the simulations was plotted for helix I (Gly6-Gly19) and helix II (Ala23-Met28) and compared between the three analogues (Fig. S4.2). Additionally, dihedral angles of residues at helix I and helix II were analyzed for the 300 K trajectory as a complementary indication of secondary structure (Fig. S4.3A-B). Surprisingly, no significant variations in helicity content and/or in the conformational preferences of the residues were observed among the three analogues. Thus, the difference in activity observed between *trans* and *cis* photorexin cannot be directly attributed to partial losses in secondary structure.

As photoisomerization was expected to affect the spatial arrangement of the two helices, we moved on to study the evolution of the intramolecular helix I - helix II angle. During REST simulations, all the peptides displayed a high degree of “hinge” flexibility which resulted in significant angle fluctuations. However, while OX-B_(6−28)_ helices were on average inclined at 45° (Fig. S4.4A), the angles in *cis* and *trans* photorexin were both closer to 90° (Fig. S4.4B-C), thus suggesting that: (i) the azobenzene moiety prevents closing of the inter-helix angle compared to the flexible hinge of the native peptide; and (ii) its photoisomerization does not further influence the angle between the two peptide fragments.

Finally, we analyzed the main clusters of conformations explored by each molecule during the 200 ns REST simulations. The most populated conformations for each derivative were locally aligned by the backbone of helix II - GILT- residues, as these constitute the hotspots for receptor binding and activation [46]. As observed from the alignment, *trans* photorexin (in green) preferentially places helix I in a similar orientation as for OX-B_(6−28)_ (in grey) (Fig. S4.5). In contrast, helix I of the *cis* isomer (in pink) is orientated in the opposite direction.

As previously demonstrated by mutagenesis studies and recently supported by cryo-EM, the α-helical N-termini of OX-B and OX_2_ as well as the ECL-2 of the latter actively engage in the formation of the receptor-peptide complex through a “message-address system” based on polytopic interactions [46, 48]. According to this model, the “address” (OX-B N-terminus) directs binding to the receptor, whereas the “message” (OX-B C-terminus) conveys the functional information [86–89]. In addition, multiple weak interactions mediate affinity towards the receptors, overall contributing to the nM potency of the peptide [48]. 

Among these hotspots for receptor binding and activation, two key residues (Leu11 and Leu15) have been identified to form a hydrophobic patch located on helix I [47, 90]. Our functional and structural data converge on attributing the lower activity displayed by *cis* photorexin to the reverse orientation adopted by its N-terminus relative to the receptor binding pocket. In particular, the UV-illuminated analogue might be unable to establish productive hydrophobic interactions, such as those predicted between the beforementioned leucine residues and Phe346 and His350 located on the extracellular region of OX_2_ (Fig. S4.6) [90, 91]. 

## Conclusions

In conclusion, we have rationally designed and developed a reliable and efficient molecular tool to photocontrol orexinergic neurotransmission. Photorexin shows that introducing a photoswitchable amino acid into the backbone of signaling peptides allows for regulating their activity in vitro and in vivo by controlling the spatial arrangement of their C- and N- terminal fragments [52]. Potentially, this approach could be extended to several endogenous peptides, that bind to their cognate receptors via a “message-address” mechanism. These include the adrenocorticotropic hormone [92], endothelins [86], and various opioid peptides [93] such as nociceptin [87, 88] and dynorphins [89] among others. Our light-regulated analogue possesses almost the same efficacy as OX-B, high nanomolar potency, and selectivity towards hOX_2_, thus showing that interventions at the flexible hinge are compatible with preserving the functional activity of the peptide. The long thermal stability and low intrinsic activity of the *cis* isomer enable administration and subsequent activation of the compound with blue light, thus offering unprecedented spatiotemporal precision to manipulate orexinergic circuits. Photorexin is the first photo-reversible ligand reported for orexin receptors. It allows dynamic control of activity in vitro and in zebrafish larvae by direct application in water. Photorexin induces dose- and light-dependent changes in locomotion and a reduction in the successive induction reflex that is associated to sleep behavior.

### Electronic supplementary material

Below is the link to the electronic supplementary material.

Supplementary Material 1

## Data Availability

Supplementary data of this article is included in the Supplementary Information file. Other data is available from the authors upon reasonable request.
