# Supplementary material for: In vivo photocontrol of orexin receptors with a nanomolar light-regulated analogue of orexin-B
Source: Cell Mol Life Sci. 2024 Jul 6;81(1):288. doi: 10.1007/s00018-024-05308-x (PMC11335211; doi:10.1007/s00018-024-05308-x)
Supplement: Supplementary file 1 — Supplementary Material 1 [file 18_2024_5308_MOESM1_ESM.docx]

SUPPLEMENTARY INFORMATION (SI)

***In vivo* photocontrol of orexin receptors with a nanomolar light-regulated analogue of orexin-B**

Davia Prischich, Rosalba Sortino, Alexandre Gomila-Juaneda, Carlo Matera, Salvador Guardiola, Diane Nepomuceno, Monica Varese, Pascal Bonaventure, Luis de Lecea, Ernest Giralt, Pau Gorostiza

Contents

[Chemical synthesis and analytical characterization 2](#_Toc169094715)

[Abbreviations 2](#_Toc169094716)

[Materials and methods 2](#_Toc169094717)

[NMR spectroscopy 2](#_Toc169094718)

[Chromatography 2](#_Toc169094719)

[Mass spectroscopy 3](#_Toc169094720)

[Synthetic procedures 3](#_Toc169094721)

[Analytical data 10](#_Toc169094722)

[Photochromic characterization 12](#_Toc169094723)

[UV-Vis absorption spectra 12](#_Toc169094724)

[Resistance to photofatigue 12](#_Toc169094725)

[Photopharmacology 13](#_Toc169094726)

[*In vitro* radioligand binding assays 13](#_Toc169094727)

[*In vitro* functional assays 14](#_Toc169094728)

[FLIPR Tetra calcium mobilization assay 14](#_Toc169094729)

[Single-cell intracellular calcium imaging 15](#_Toc169094730)

[*In vivo* functional assays 17](#_Toc169094731)

[Structural characterization 20](#_Toc169094732)

[Circular dichroism 20](#_Toc169094733)

[Replica exchange molecular dynamics (REMD) 20](#_Toc169094734)

[Additional references 26](#_Toc169094735)

# Chemical synthesis and analytical characterization

## Abbreviations

ACN: acetonitrile; AcOH: acetic acid; Ac_2_O: acetic anhydride; AMPP: [3-(3-aminomethyl)phenylazo]phenylacetic acid; DCM: dichloromethane; DIC: *N*,*N*′-diisopropylcarbodiimide; DIPEA: *N*,*N*-diisopropylethylamine; DMF: dimethylformamide; DMSO: dimethylsulfoxide; equiv: equivalent; EtOAc: ethyl acetate; EtOH: ethanol; Et_2_O: diethyl ether; FA: formic acid; Fmoc-OSu: 9-fluorenylmethylsuccinimidyl carbonate; HATU: 1-[bis(dimethylamino) methylene]-1*H*-1,2,3-triazolo[4,5-*b*]pyridinium 3-oxid hexafluorophosphate; MeOH: methanol; mQ: milliQ; rpm: revolutions per minute; rt: room temperature; *R*t: retention time; TFA: trifluoroacetic acid; TIS: triisopropylsilane.

## Materials and methods

All reagents and solvents were purchased from commercial suppliers and were used without any further purification. Analytical thin layer chromatography (TLC) was performed on silica gel 60 F254 aluminum foils (Merck Millipore, Darmstadt, DE). Spots were visualized under 254 or 365 nm UV light and/or by using the appropriate TLC stain. Flash chromatography was performed on silica gel 60 (40-63 μm) as stationary phase (PanReac AppliChem, Darmstadt, DE).

## NMR spectroscopy

Samples for nuclear magnetic resonance (NMR) were dissolved in Chloroform-*d*. Spectra were recorded with a Varian Mercury 400 MHz instrument (400 MHz for ^1^H-NMR) and referenced to tetramethylsilane and/or residual solvent peaks (Chloroform-*d*: ^1^H 7.26 ppm). Chemical shifts (δ) are expressed as parts-per-million (ppm) and they refer to the *trans* isomers unless differently stated. Coupling constants (*J*) are measured in hertz (Hz). Spectra were analysed using MestReNova Lite CDE (Mestrelab Research S.L., Santiago de Compostela, ES).

## Chromatography

**HPLC-MS**

High-pressure liquid chromatography (HPLC) coupled to mass spectrometry (MS) was performed on a Waters Alliance e2695 Separations Module equipped with an automatic injector, a Waters 2998 UV-Vis Photodiode Array Detector (PDA) and a QDA Detector II (Waters, Milford, MA). Samples were solved in MeOH and filtered through a 0.22 μm filter before being injected on a SunFire® C18 column (100 Å, 5 µm, 150 x 4.6 mm). The mobile phase was a mixture of mQ H_2_O and ACN supplemented with 0.1% FA (v/v). Analysis were carried out at rt applying a 0 to 100% 8-min linear gradient while keeping the flow rate at 1 ml/min. Data were acquired using the MassLynx™ 4.1 software.

**UPLC and UPLC-MS**

Ultra-high pressure liquid chromatography (UPLC) and UPLC coupled to mass spectrometry (UPLC-MS) were performed with an ACQUITY UPLC H-Class System (Waters, Milford, MA) equipped with a Flow Through Needle-Sample Manager (SM-FTN), column heater, quaternary solvent manager and an ACQUITY UPLC PDA eλ Detector. UPLC-MS mass spectra were obtained with a SQ Detector2 (Waters) equipped with an electrospray ionization (ESI) interface. Empower™ 3 and MassLynx™ 4.1 were respectively used as softwares for data acquisition. Analysis were run on an ACQUITY BEH C18 (130 Å, 1.7 µm, 50 x 2 mm) column, which was maintained at 40 ˚C. The mobile phase was a mixture of mQ H_2_O and ACN respectively supplemented with either 0.045% and 0.036% TFA (v/v) for UPLC analysis or 0.1% and 0.07% FA (v/v) for UPLC-MS. The flow rate was maintained at 0.6 ml/min while applying 25 to 50% 2-min long linear gradients. Peptide samples were prepared by dissolving the solid in mQ H_2_O and then filtered through a 0.22 μm filter before injection. Detection was performed at 220 and 323 nm.

## Mass spectroscopy

High-resolution mass spectrometry (HRMS) was performed at the IRB Barcelona Mass Spectrometry and Proteomics Core Facility with an LTQ-FT Ultra Mass Spectrometer (Thermo Scientific, Waltham, USA) using automated positive Nano-Electrospray Ionization (NanoESI) for direct infusion. The compound was dissolved in mQ H_2_O (135 µM) and diluted 1:100 with ACN/H_2_O/FA (50:50:1) for MS analysis. The NanoMate (Advion BioSciences, Ithaca, USA) aspirated the sample from a Protein LoBind 384-well plate using disposable, conductive pipette tips. The sample was infused through the NanoESI Chip (400 nozzles in a 20x20 array) towards the mass spectrometer. Spray voltage was 1.70 kV and delivery pressure was 0.50 psi. Data was acquired with Xcalibur software, vs.2.0SR2 (Thermo Scientific). Data are reported as mass-to-charge ratios (*m*/*z*) of the corresponding positively charged molecular ions. Ion deconvolution to zero-charged monoisotopic masses was performed using Xtract algorithm in Xcalibur software and elemental compositions from experimental exact mass monoisotopic values were obtained with a dedicated algorithm integrated in Xcalibur software.

## Synthetic procedures

**Synthesis of Fmoc-AMPP-OH**

The AMPP photoswitchable amino acid was synthesized adapting a protocol published by Podewin *et al*.^1^

**Scheme S1.** Route to Fmoc-AMPP-OH [3-(3-aminomethyl)phenylazo]phenylacetic acid (AMPP) adapted from Dong *et al*.^2^ a) Fmoc-OSu, Et_3_N, rt; b) NH_4_Cl, Zn powder, rt, Fe_3_Cl; c) AcOH, rt

**Synthesis of (9*H*-fluoren-9-yl)methyl (3-aminobenzyl)carbamate (2)**

Fmoc-OSu (7.0 g, 22.0 mmol, 1 equiv.) was dissolved in 50 ml ACN and slowly added to a solution of 3-(aminobenzyl)amine (2.21 ml, 22.0 mmol, 1 equiv.), 3.0 ml (22.0 mmol, 1 equiv.) Et_3_N and 27.5 ml of an ACN/DMF (10:1) mixture. The mixture was stirred for 4 h at rt and quenched with 25 ml mQ H_2_O. The resulting precipitate was filtered off, washed with 25 ml *tert*-butyl methyl ether /trifluoroethanol (1:1) and dried under high vacuum to give the titled compound as a white solid (3.90 g, 52%). The product was used in the next reaction without further purification. Spectra conformed to those previously described in the literature.^1^

^1^H-NMR (400 MHz, Chloroform-*d*): δ 7.77 (d, *J* = 7.2 Hz, 2H), 7.60 (d, *J* = 7.4 Hz, 2H), 7.40 (t, *J* = 7.3 Hz, 2H), 7.31 (t, *J* = 7.3 Hz, 2H), 7.12 (t, *J* = 7.6 Hz, 1H.), 6.65 (d, *J* = 7.6 Hz, 1H), 6.60 (m, 3H), 5.03 (bs, 1H), 4.45 (d, *J* = 6.9 Hz, 2H), 4.30 (d, *J* = 5.8 Hz, 2H), 4.24 (t, *J* = 7.0 Hz, 1H).

**Synthesis of 2-(3-nitrosophenyl)acetic acid (4)**

A solution of 2-(3-nitrophenyl)acetic acid (2.50 g, 13.2 mmol, 1.0 equiv.) in 100 ml 2-methoxyethanol was stirred for 10 min at rt under an argon atmosphere. Subsequently, a solution of NH_4_Cl (1.09 g, 20.3 mmol, 1.5 equiv.) in 25 ml mQ H_2_O was added to the mixture. Upon cooling the solution to 0 °C, zinc (2.10 g, 32.3 mmol, 2.5 equiv.) was slowly added to the mixture within 40 min. After 1 h, the reaction was filtered and the filtrate was added to a solution of FeCl_3_ ∙ 6 H_2_O (11.2 g, 41.4 mmol, 3.0 equiv.) in 120 ml EtOH/H_2_O (2:1) while maintained at 0 °C in an ice bath. The reaction was allowed to stand for 1.5 h, before being diluted with mQ H_2_O (200 ml) and extracted with Et_2_O (3x100 ml). The organic layers were combined, dried over anhydrous MgSO_4_, and brought to dryness under reduced pressure. The crude was purified by column chromatography on silica gel (eluent EtOAc/cyclohexane 90:10 + 0.5% acetic acid). The product was afforded as a dark oil (1.65 g, 75% yield). Spectra matched those previously reported in the literature.^1^

^1^H-NMR (400 MHz, Chloroform-*d*): δ = 8.16 (d, *J* = 7.5 Hz, 1H), 7.88 (d, *J* = 7.3 Hz, 1H), 7.76 (s, 1H), 7.63 (t, *J* = 7.5 Hz, 1H), 3.82 (s, 2H).

**Synthesis of [3-(3-aminomethyl)phenylazo]phenylacetic acid (5)**

To a solution of **4** (0.54 g, 2.3 mmol, 1 equiv.) in 16 ml glacial acetic acid, 1.14 g of **2** (2.3 mmol, 1 equiv.) was added portionwise. The resulting mixture was stirred for 24 h at rt before removing the solvent under reduced pressure. The crude was purified through flash chromatography on silica gel (EtOAc/cyclohexane 50:50 + 0.1% acetic acid). The product was obtained as an orange solid (0.76 g, 59%). Spectra conformed to those previously described in the literature.^1^

HPLC purity: 98% (**Figure S1.1**)

*R*t (HPLC): 4.73 min (*cis* isomer) 5.47 min (*trans* isomer) (**Figure S1.1**)

^1^H-NMR (400 MHz, Chloroform-*d*): δ 7.84 (m, 4H), 7.75 (d, *J* = 7.4 Hz, 2H), 7.60 (d, *J* = 7.1 Hz, 2H), 7.48 (t, *J* = 8.3 Hz, 2H), 7.39 (m, 4H), 7.30 (t, *J* = 7.6 Hz, 2H) 5.22 (bs, 1H), 4.49 (m, 4H), 4.24 (t, *J* = 6.9 Hz, 1H), 3.75 (s, 2H). (**Figure S1.2**)


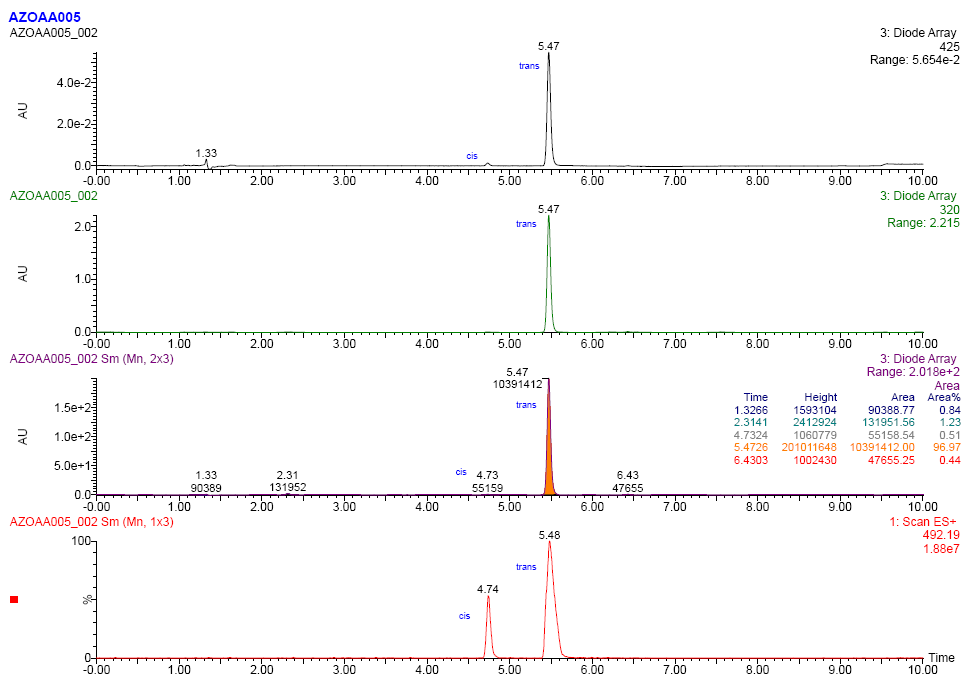


**Figure S1.1** HPLC-MS analysis of AMPP.


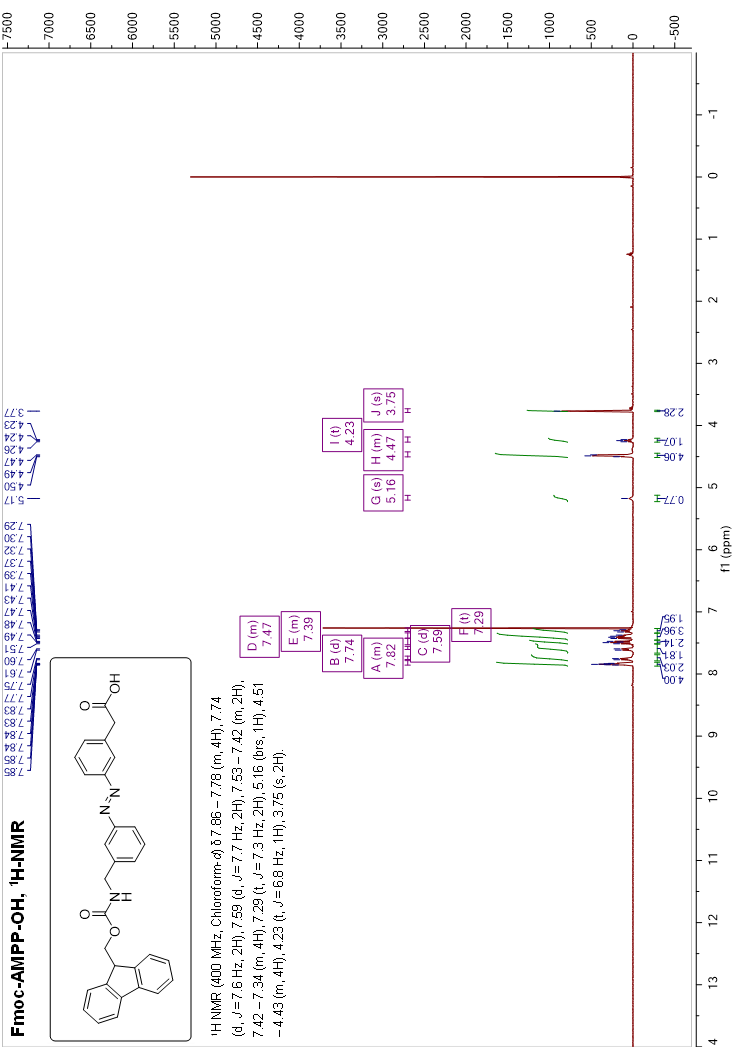


**Figure S1.2** ^1^H-NMR of AMPP.

**Peptide synthesis and purification**

The peptide was synthesized by standard 9-fluorenylmethyloxycarbonyl (Fmoc) solid-phase peptide synthesis (SPPS) on a 0.1 mmol scale. Peptide elongation was performed using an automated microwave-assisted peptide synthesizer combined with subsequent manual elongation of the chain. Manipulation of the peptide for the manual synthesis was done in polypropylene syringes with a polypropylene porous filter (Scharlau, ES). Periodic stirring was done manually with a Teflon stirring bar, while solvents and soluble reagents were removed by vacuum filtration. Washings between synthetic steps were done with dimethylformamide (DMF, 5 x 30 s), dichloromethane (DCM, 5 x 30 s), and then DMF again (5 x 30 s) using 10 ml of solvent/g resin each time.

Protected L-amino acids (Iris Biotech, Marktredwitz, DE) used for the Fmoc chemistry were the following unless otherwise specified: Fmoc-Ala-OH∙H_2_0, Fmoc-Gly-OH, Fmoc-Ile-OH, Fmoc-Leu-OH, Fmoc-Met-OH, Fmoc-Gln(Trt)-OH, Fmoc-Arg(Pbf)-OH, Fmoc-Ser(*t*Bu)-OH, Fmoc-Thr(*t*Bu)-OH and Fmoc-AMPP-OH.

**Resin conditioning and loading of the first amino acid**

A RinkAmide ChemMatrix® resin (PCAS BioMatrix Inc., Saint Jean sur Richelieu, CA) with a substitution of 0.54-0.56 mmol/g was used as solid support. Prior to the first amino acid coupling, the resin was swelled in DCM for 30 minutes and subsequently conditioned as follows:

**Table S1.1** Resin conditioning

| **Treatment** | **Reagents** | **Time** |
| --- | --- | --- |
| Washing | MeOH, DMF, DCM | 5 x 30 s for each solvent |
| Washing | 1% TFA in DCM | 1 x 1 min, 2 x 10 min |
| Washing | DCM, DMF, DCM | 5 x 30 s for each solvent |
| Washing | 5% DIPEA in DCM | 1 x 1 min, 2 x 10 min |
| Washing | DCM, DMF, DCM | 5 x 30 s for each solvent |

In order to quantify the loading capacity of the resin, the first amino acid was inserted manually. Coupling and recoupling were performed following conditions as summarized in **Table S1.2**. Finally, unreacted amines still present on the resin were acetylated to avoid formation of deletion sequences. Between each step, the mixture was washed with DMF, DCM, and DMF again (5 x 30 s for each solvent).

**Table S1.2** Loading of the first amino acid to the resin

| **Treatment** | **Reagents** | **Conditions** |
| --- | --- | --- |
| Washing | DMF, DCM, DMF | 5 x 30 s for each solvent |
| Coupling | 4 equiv. Fmoc-Met-OH, 4 equiv. DIC, 4 equiv. Oxyma | DMF, 1 h, 100 rpm |
| Washing | DMF, DCM, DMF | 5 x 30 s for each solvent |
| Recoupling | 4 equiv. Fmoc-Met-OH, 3.9 equiv. HATU, 8 equiv. DIPEA | DMF, 40 min, 100 rpm |
| Washing | DMF, DCM, DMF | 5 x 30 s for each solvent |
| Capping | 10 equiv. Ac_2_O, 20 equiv. DIPEA | DMF, 15 min |
| Washing | DMF, DCM, DMF | 5 x 30 s for each solvent |

**Fmoc deprotection and resin loading quantification**

The Fmoc group was removed by treating the resin with 20% (v/v) piperidine in DMF (10 ml/g resin - 1 x 1 min, 2 x 10 min). In order to quantify the resin functionalization, piperidine washes were collected in a volumetric flask, brought to volume with DMF and the absorbance was measured at 300 nm by UV spectroscopy. Loading of the resin was calculated using the following equation:

*f* $=$ $\frac{A\cdot V}{\varepsilon\cdot l\cdot m}$

where *f* is the degree of functionalization, *A* is the measured absorbance at 300 nm, *V* is the volume of the volumetric flask in ml, 𝜀 is the molar extinction coefficient of Fmoc at 300 nm (7800 M^-1^cm^-1^), *l* is the optical path (1 cm), and *m* the mass of the resin in mg.

**Coupling methods**

**Automated microwave-assisted peptide synthesis**

A CEM Liberty Blue microwave peptide synthesizer was used. Drain washings were performed with DMF (2 x 5 ml) with nitrogen gas agitation. The same protected amino acids as for the manual synthesis were used.

The C-terminus of photorexin (AAGILTM-NH_2_) was synthesized using this method. Fmoc-AAs-OH (0.2 M in DMF) were coupled in a 5-fold excess in the presence of DIC (5 equiv., 1.5 M in DMF) and Oxyma Pure (5 equiv., 1.5 M in DMF). The mixtures were allowed to react for 3 min at 90 ˚C (potency 35 W). Fmoc deprotection was carried out during 1 min at 90 ˚C (potency 35 W) using 10% (w/v) piperazine and 0.1 M Oxyma Pure in a 9:1 mixture of *N*-methylpyrrolidinone and EtOH. After the final Fmoc-deprotection the resin was washed with DMF (2 x 10 ml) and moved to a polypropylene syringe for manual incorporation of the remaining amino acids.

**Manual synthesis**

The method illustrated in **Table 1.3** was used as a default to incorporate all Fmoc-AAs-OH to the peptidyl resin. If from the Kaiser test the first coupling resulted incomplete, a second coupling was performed repeating the same conditions. Fmoc deprotection was carried out with 20% (v/v) piperidine in DMF (10 ml/g resin - 2 x 10 min). All steps were performed protecting the resin from direct light.

**Table 1.3** - Standard protocol for manual Fmoc-AA-OH incorporation

| **Step** | **Treatment** | **Reagents** | **Duration** |
| --- | --- | --- | --- |
| 1. | Washing | DMF | 5 x 30 s |
| 2. | Deprotection | 20% (v/v) piperidine in DMF | 2 x 10 min |
| 3. | Washing | DMF | 5 x 30 s |
| 4. | Washing | DCM | 5 x 30 s |
| 5. | Test ^a^ | Ninhydrin test | 3 min at 110 ˚C |
| 6. | Washing | DMF | 5 x 30 s |
| 7. | Coupling ^b^ | 4 equiv. Fmoc-AA-OH,  4 equiv. DIC, 4 equiv. Oxyma  in 1 ml DCM/DMF (1:1) | 45 min |
| 8. | Washing | DMF | 5 x 30 s |
| 9. | Washing | DCM | 5 x 30 s |
| 10. | Test ^a,c^ | Ninhydrin test | 3 min at 110 ˚C |

^a^ If negative: go to step 1; ^b^ Repeat if step 10 gives positive result; ^c^ If positive: go to 7.

**Monitoring of deblocking and coupling**

A Kaiser or ninhydrin test was performed after each deprotection step to verify Fmoc removal and after each coupling to check if the new protected amino acid had been successfully incorporated into the chain. In addition to this, a test cleavage was performed every 4-6 amino acids on a small quantity of resin. The growing chain was monitored by UPLC-MS.

**Cleavage and sidechains deprotection**

After Fmoc removal from the N-terminus of the peptide, the resin was washed thoroughly with DMF and DCM and then dried under vacuum for 15 min. The dried resin was transferred to a 50 ml falcon and the peptide was cleaved using a cocktail containing TFA, H_2_O, and TIS (95:2.5:2.5).

The peptide was left to react for 2 h using enough solvent to allow a good swelling of the resin. After cleavage of the peptide, the solvent was evaporated applying a N_2_ current. The solid was washed at least 3 times by suspension in *tert*-butyl methyl ether stored at -20 °C, and subsequent centrifugation. After that, the cleaved peptide was dissolved in mQ H_2_O/ACN (1:1) and freeze-dried.

**Purification**

The crude peptide was dissolved in mQ H_2_O and filtered through a 0.22 μm filter before purification by analytical HPLC. The system consisted of a Waters Alliance e2695 Separations Module equipped with an automatic injector and a Waters 2998 UV/Vis Photodiode Array Detector (Waters, Milford, MA). Samples (between 20 and 100 µl) were injected on a SunFire® C18 column (100 Å, 3.5 µm, 100 x 4.6 mm). The mobile phase was a mixture of mQ H_2_O and ACN respectively supplemented with 0.045% and 0.036% TFA (v/v). Purification was carried out at rt applying a 30 to 50% 8-min linear acetonitrile gradient while keeping the flow rate at 1 ml/min. Data were acquired using the Empower™ 3 software. To allow manual collection of the fractions, the waste tube coming out from the detector was replaced with a short rigid outlet. The eluate was collected at the appropriate retention time (*R*t), while monitoring the run at both 220 and 323 nm. The corresponding fractions were then pooled together and lyophilized. The purity of the final product was analysed by UPLC and UPLC-MS as previously described.

## Analytical data

UPLC purity: 98% (**Figure S1.3**)

*R*t (UPLC): 1.68 min (*trans* isomer), 1.76 min (*cis* isomer) (**Figure S1.3**)

HR-MS (ESI): *m/z* calculated for C_107_H_180_N_34_O_27_S_1_^2+^ [M+2H]^2+^: 1202.6660; found: 1203.1745. Deconvoluted spectrum C_107_H_178_N_34_O_27_S_1_ [M]: 2403.33214; found: 2403.33216. (**Figure S1.4**)

**Figure S1.3** UPLC chromatogram of pure phOX as obtained under benchtop conditions.

**Figure S1.4** Full high-resolution and deconvoluted monoisotopic mass spectra of phOX.

# Photochromic characterization

## UV-Vis absorption spectra

**Figure S2.1** Absorption spectra of phOX in mQ H_2_O (20 µM, 25 °C) in the dark-adapted state (dashed line) and after cumulative exposure to illumination with a UVGI torch light.

## Resistance to photofatigue

**Figure S2.2** Reversibility and stability of the photochromic behavior of phOX (25 µM in PBS at pH 7.4 and 25 °C) over several cycles (3 minutes each) of photoinduced isomerization at 365 nm (*trans→cis*) and 455 nm (*cis→trans*). Integration areas of the peaks corresponding to the *trans* and *cis* isomers were determined by UPLC analysis after each cycle. Empower™ 3 reports peak area as µV∙second and the UV detector was set so that 1 V = 1 AU meaning that a 1-AU peak is equivalent to a peak height of 1,000,000 height counts from a baseline of 0 V.

# Photopharmacology

## *In vitro* radioligand binding assays

The affinity towards OX_2_ was studied in a competitive radioligand binding assay on membranes derived from HEK-293 cells stably transfected with the human receptor. [^3^H]EMPA, an OX_2_-selective orthosteric antagonist, was used as radioactive tracer to quantify residual binding after competitive displacement in the presence of the unlabeled ligands.^3^ Wild-type OX-B and almorexant, a non-selective OX_1_ and OX_2_ antagonist, served as controls.^4^

**Drugs**

[^3^H]EMPA (specific activity 27 Ci/mmol) and almorexant were synthesized at Janssen Research & Development LLC (San Diego, CA), while orexin-B was obtained from Bachem (Torrance, CA).

**Radioligand** **competitive** **binding** **assay**

Cell membranes were prepared from a stable pool of HEK-293 cells transfected with the human OX_2_. Test compounds were dissolved in DMSO (10 mM stock solutions) and diluted in Dulbecco’s phosphate-buffered saline supplemented with 0.1% bovine serum albumin (BSA). Competition studies were performed by incubating the membranes with the OX_2_-selective antagonist [^3^H]EMPA (2 nM) and different concentrations of the test compounds (7 concentrations ranging from 10^−9^ to 10^−6^ M). After a 60-minute incubation at rt, binding reactions were filtered to separate the excess of free ligand from the membranes. Filters were then counted for radioactivity in a scintillation counter. Nonspecific binding was determined in the presence of 10 µM almorexant. Data from competition binding experiments were normalized to total (100%) and unspecific binding (0%) and are displayed as means ± SD from two independent experiments.

**Data analysis and statistics**

Specific binding was determined and plotted as a function of the concentration of the compound using GraphPad Prism v8.3.1 (GraphPad Software, San Diego, USA). Data were analyzed by nonlinear regression using a one-site competition model.

**Results and discussion**

As shown in **Figure S3.1** for a wide concentration range (nM-µM), photorexin *trans* and *cis* isomers did not inhibit [^3^H]EMPA binding to the receptor, as neither did the native peptide. On the other hand, the positive control – almorexant – showed great affinity and fully displaced the radioligand. Its inhibition constant was calculated by nonlinear regression using a one-site competition model and the resulting value proved consistent with literature data (Ki = 3 nM).^3^

Although the lack of binding with all three forms of OX-B - including the native ligand control - came unexpected, similar findings had already been observed for the OX_1_ receptor. In a membrane-based binding assay reported by Langmead *et al.*, both OX-A and OX-B failed to occupy the receptor orthosteric site when in competition with a small-molecule antagonist ([^3^H]SB-674042).^5^ As GPCRs exist in different conformations, agonist peptides might bind only to the fraction of receptors that are coupled to G proteins (active state displaying high agonist affinity), whereas antagonists can occupy all the sites independently of the receptor being active or inactive.^6,7^ Being binding results inconclusive, we moved to directly assess phOX activity in an *in vitro* functional assay.

**Figure S3.1** Specific binding curves showing the displacement of [^3^H]EMPA, an OX_2_-selective antagonist, in membrane preparations from HEK-293-hOX_2_. Almorexant (in blue) showed high affinity and fully displaced the radioligand, whereas all three forms of OX-B, wild-type (WT, in grey), dark-adapted phOX (*trans*, in green) and UV-illuminated phOX (*cis*, in pink) did not compete to bind to the receptor. Data are means ± SD of two independent experiments run in triplicates.

## *In vitro* functional assays

### FLIPR Tetra calcium mobilization assay

**Figure S3.2** OX-B (wild-type, in grey), dark-adapted phOX (*trans*, in green) and pre-irradiated phOX (*cis*, in pink) evoke a concentration-dependent increase in intracellular [Ca^2+^]. [Ca^2+^]_i_ was monitored using the FLIPR Tetra system in CHO-K1 cells stably expressing hOX_1_ (left panel) or in PFSK-1 cells, a cell line that innately express hOX_2_ (right panel). *Cis* phOX was pre-illuminated during 10 min with 365 nm UV light before testing. Responses were measured as maximum increase in fluorescence minus basal and are given as means ± SD from two independent experiments run in triplicates.

**Table S3.1** *In vitro* functional potencies, efficacies, and calculated receptor selectivity of OX-B and dark-adapted phOX (*trans*) at hOX_1_ and hOX_2_ receptors. Note that the indicated efficacy results from the mathematical fit to a dose-response curve of cell responses measured with a non-linear fluorescent calcium indicator.


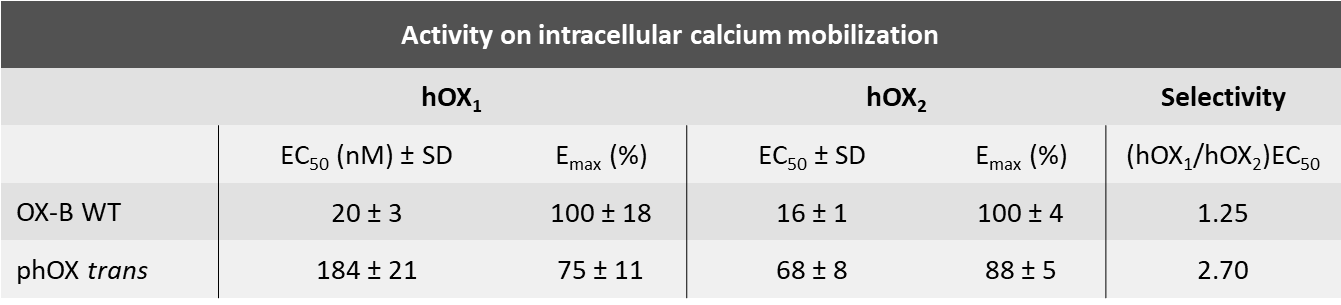


### Single-cell intracellular calcium imaging

**Figure S3.3** **A**) *In vitro* calcium imaging with tsA201 cells expressing the genetically encoded calcium indicator R-GECO1 but not transfected with hOXs. No calcium oscillation was observed after application of 100 nM phOX in the dark-adapted state or during concomitant illumination at 365 and 455 nm, thus discarding artifacts (*n*=20). Gray shading area represents standard error of the mean (SEM). **B**) TsA201 cells transfected with GFP-labelled hOX_2_ (in green) and R-GECO1 (in red). Co-transfected cells that can be used for calcium imaging quantification appear in orange (merge).

**Figure S3.4 A**) Representative single-cell calcium traces (*n*=10) in tsA201 cells transiently expressing GFP-hOX_2_ and R-GECO1. Upon application of 30 nM photorexin (green bar) in its dark-adapted state (*trans,* white area) cells gave sharp responses. Calcium oscillation were then abolished upon exposure to cycles of UV light (365 nm, *cis*-enriched state in pink) and recovered with blue illumination (455 nm, *trans*-enriched state in blue). **B**) Real-time intracellular calcium recordings (averaged traces, black line, *n*=20) from tsA201 cells co-expressing hOX_2_ and R-GECO1 upon application of 30 nM photorexin (green bar). Traces were recorded in the dark (white area) and under cycles of illumination with UV (365 nm, pink) and blue (455 nm, blue) light. Grey dotted band represents SEM.

**Figure S3.5** Dose-response curves of dark-relaxed (in green), UV-illuminated (365 nm, in pink), and back-isomerized (455 nm, in blue) phOX at the hOX_1_ and hOX_2_ receptors (dashed and solid curves, respectively). Data are means ± SEM of two independent experiments.

## *In vivo* functional assays

**Figure S4.1** Traces representing fast-swimming distances (faster than 6 mm·s^-1^) of larvae treated with vehicle (in black) or 1 µM phOX (in blue). Distances were integrated over 1-min intervals. Patterned areas represent ± SEM (*n*=12 larvae per condition).

**Figure S4.2** Magnification of the traces from **Figure 4.A** under the alternating UV and visible illumination. Fast-swimming distances (faster than 6 mm·s^-1^) are plotted for larvae treated with different doses of phOX (vehicle – black, 10 nM – yellow, 100 nM – green, 1 µM – blue, and 10 µM – red traces). Distances were integrated over 1-min intervals. Patterned areas represent ± SEM (*n*=12 larvae per condition).

**Figure S4.3** Detail of the traces from **Figure 4.A** including vehicle (0 nM, in black), 10 nM (yellow), 50 nM (orange), 100 nM (green), 500 nM (purple), 1 µM (blue), and 10 µM (red) photorexin. Note that traces for 50 nM and 500 nM are not shown in **Figure 4.A** for clarity, but their quantification is included in **Figure 4.B**.

**Figure S4.4** Dose-response curves in zebrafish larvae treated either with vehicle or with different concentrations of photorexin in the dark (resting period - RP, black), under 365 nm (in purple), and 455 nm (in blue) light. Normalized swum distances were measured in mm upon integrating the time course traces between minutes 16 and 20 for the RP in the dark or the 3 consecutive 1-min illumination intervals between minutes 20 and 26 for each wavelength (365 nm and 455 nm). The time course traces are shown in **Figures 4A** and **S4.1-S4.3**. Data at 0 nM corresponds to the vehicle under each illumination condition. During the RP (black curve), dark-relaxed photorexin produces increased locomotion at concentrations 500 nM, 1 µM, and 10 µM, but the differences with 365 nm illumination (purple trace) are still significant. Patterned areas represent ± SD (*n* = 12 larvae per treatment group). Statistical differences for 365 nm  (purple stars) and RP  (black stars) were determined by two-way ANOVA with Dunnett’s multiple comparison test (*, *p*-value ≤ 0.05; ***, *p*-value ≤ 0.001; ****, *p*-value ≤ 0.0001). Illumination at 455 nm showed no significant effect for all concentrations.

# Structural characterization

## Circular dichroism

**Figure S4.1** Circular dichroism spectra of phOX in PBS (**A**, solid traces) and PBS supplemented with 30% trifluoroethanol (**B**, dashed traces) at pH 7.4 and 28 °C. Spectra were recorded in the dark-adapted state (green traces) and after illumination at 365 nm (pink traces) and normalized as mean residue ellipticity [Θ]_MRE_.

## Replica exchange molecular dynamics (REMD)

**Figure S4.2** Average number of H-bonds during the 300 K simulation between backbone atoms comprising the helix II (Ala23-Met28, solid bars) and helix I (Gly6-Gly19, dashed bars). No significant differences in helicity are observed between the three analogues. Data represent means ± SD. Statistical analysis was performed by Student’s *t* test.

**Figure S4.3A** Dihedral angles of residues at helix I (Gln8-Glu16) and helix II (Ala23-Thr28) observed for truncated OX-B, *cis* phOX and *trans* phOX during the 300 K simulation.

**Figure S4.3B** Dihedral angles of residues at helix I (Gln8-Glu16) and helix II (Ala23-Thr28) observed for truncated OX-B, *cis* phOX and *trans* phOX during the 300 K simulation.

**Figure S4.4** Evolution of the intramolecular helix I-helix II angle of OX-B_(6-28)_ (**A**), *trans* (**B**) and *cis* (**C**) phOX during the REST simulation. Trajectories of each frame are shown in light blue, whereas the overall average is highlighted in orange.


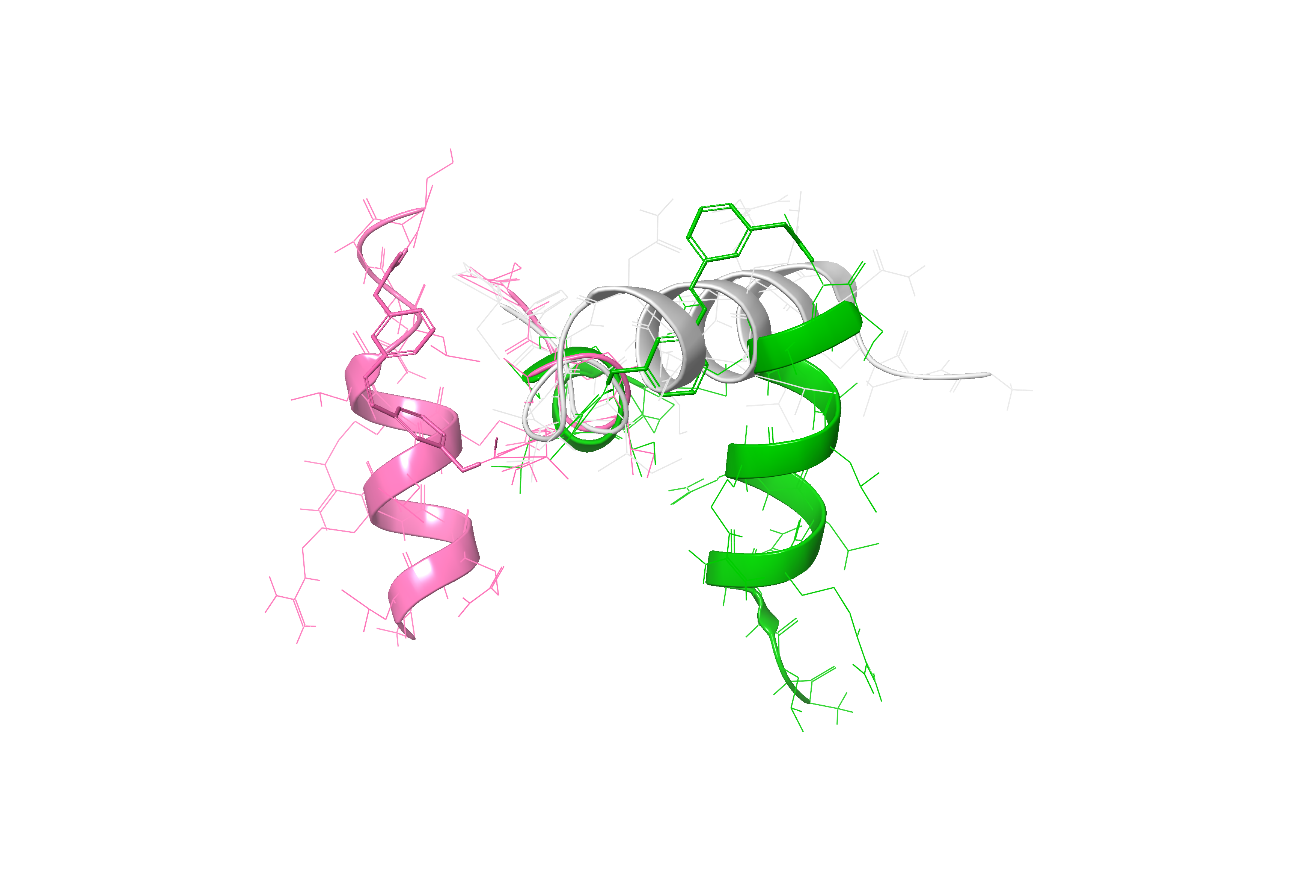


**Figure S4.5** Most populated conformations for OX-B_(6-28)_ (in grey), and *trans* and *cis* phOX (represented in green and pink, respectively) obtained from REMD simulation. GILT- residues in helix II were aligned to compare the preferential orientation of helix I in the three derivatives.

**Figure S4.6** **A**) X-ray structure of the extracellular domain of OX_2_ (PDB ID: 4S0V) with Phe346 and His350 showed in red.^8^ These residues are key hotspots for anchoring the peptide to the receptor pocket by means of hydrophobic interactions with helix I residues Leu11 and Leu15.^9,10^ **B**) Looking from the top, when the three peptides are aligned on the receptor binding site, there is a similar orientation of OX-B_(6-28)_  and the *trans* isomer of photorexin (towards the right-hand side of the figure, with peptide residues Leu11 and Leu15 facing the receptor residues Phe346 and His350)*,* while the *cis* peptide is oriented towards the opposite region of the receptor (leftwards in the figure, non-interacting with the receptor residues Phe346 and His350).

# Additional references

1. Podewin, T. *et al.* Photocontrolled chignolin-derived β-hairpin peptidomimetics. *Chem. Commun.* **51**, 4001–4004 (2015).

2. Dong, S. L. *et al.* A photocontrolled β-hairpin peptide. *Chem. - A Eur. J.* **12**, 1114–1120 (2006).

3. Malherbe, P. *et al.* Biochemical and behavioural characterization of EMPA , a novel high-affinity, selective antagonist for the OX 2 receptor. *Br. J. Pharmacol.* **156**, 1326–1341 (2009).

4. Malherbe, P., Borroni, E., Pinard, E., Wettstein, J. G. & Knoflach, F. Biochemical and electrophysiological characterization of almorexant, a dual orexin 1 receptor (OX1)/orexin 2 receptor (OX2) antagonist: Comparison with selective OX1 and OX2 antagonists. *Mol. Pharmacol.* **76**, 618–631 (2009).

5. Langmead, C. J. *et al.* Characterisation of the binding of [ 3H]-SB-674042, a novel nonpeptide antagonist, to the human orexin-1 receptor. *Br. J. Pharmacol.* **141**, 340–346 (2004).

6. Rosenbaum, D. M., Rasmussen, S. G. F. & Kobilka, B. K. The structure and function of G-protein-coupled receptors. *Nature* **459**, 356–363 (2009).

7. S.-H. Park, P. Ensemble of G Protein-Coupled Receptor Active States. *Curr. Med. Chem.* **19**, 1146–1154 (2012).

8. Yin, J., Mobarec, J. C., Kolb, P. & Rosenbaum, D. M. Crystal structure of the human OX2 orexin receptor bound to the insomnia drug suvorexant. *Nature* **519**, 247–250 (2015).

9. Malherbe, P. *et al.* Mapping the Binding Pocket of Dual Antagonist Almorexant to Human Orexin 1 and Orexin 2 Receptors: Comparison with the Selective OX 1 Antagonist SB-674042 and the Selective OX 2 Antagonist N -Ethyl-2-[(6-methoxy-pyridin-3-yl)-(toluene-2-sulfonyl)-amino]- . *Mol. Pharmacol.* **78**, 81–93 (2010).

10. Heifetz, A. *et al.* Toward an understanding of agonist binding to human orexin-1 and orexin-2 receptors with G-protein-coupled receptor modeling and site-directed mutagenesis. *Biochemistry* **52**, 8246–8260 (2013).
